# Damming an electronic energy reservoir: ion-regulated electronic energy shuttling in a [2]rotaxane†

**DOI:** 10.1039/d1sc02225c

**Published:** 2021-06-04

**Authors:** Shilin Yu, Arkady Kupryakov, James E. M. Lewis, Vicente Martí-Centelles, Stephen M. Goldup, Jean-Luc Pozzo, Gediminas Jonusauskas, Nathan D. McClenaghan

**Affiliations:** Institut des Sciences Moléculaires, University of Bordeaux/CNRS Talence France nathan.mcclenaghan@u-bordeaux.fr; Laboratoire Ondes et Matière d'Aquitaine, University of Bordeaux/CNRS Talence France; School of Chemistry, University of Southampton Highfield Southampton SO17 1BJ UK s.goldup@soton.ac.uk; Department of Chemistry, University of Jyvaskyla 40014 Jyväskylä Finland; Department of Chemistry, Imperial College London, Molecular Sciences Research Hub 82 Wood Lane London W12 0BZ UK

## Abstract

We demonstrate the first example of bidirectional reversible electronic energy transfer (REET) between the mechanically bonded components of a rotaxane. Our prototypical system was designed such that photoexcitation of a chromophore in the axle results in temporary storage of electronic energy in a quasi-isoenergetic “reservoir” chromophore in the macrocycle. Over time, the emissive state of the axle is repopulated from this reservoir, resulting in long-lived, delayed luminescence. Importantly, we show that cation binding in the cavity formed by the mechanical bond perturbs the axle chromophore energy levels, modulating the REET process, and ultimately providing a luminescence read-out of cation binding. Modulation of REET processes represents an unexplored mechanism in luminescent molecular sensor development.

## Abstract 2

Delayed emission due to reversible electronic energy transfer (REET) between chromophores in the axle and macrocycle components of a rotaxane is demonstrated. The REET process can be modulated by metal ion binding in the cavity of the rotaxane.

## Graphical abstract

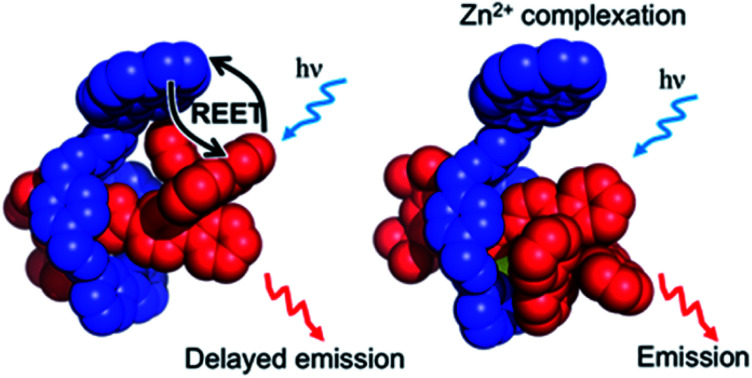

## Introduction

Luminescent rotaxanes^1^ in which the mechanical bond^2^ between a linear axle and an encircling macrocycle alters the properties of a luminophore in either component have been developed for a range of purposes, including monitoring the relative co-conformation of the sub-units,^3^ sensing of guests bound within the cavity of the rotaxane,^4^ and improving the stability^5^ and emission properties^6^ of the luminophore itself. Light has also been successfully used to promote processes in interlocked structures such as topological transformations^7^ dethreading/threading,^8^ and shuttling.^9^ The mechanical bond has also been used to study or optimize photophysical processes that rely on electronic energy^10^ (*e.g.* quenching, FRET), or electron transfer^11^ between molecular sub-units by taking advantage of the fact that, although there is no covalent bond between the components of a rotaxane, they are unable to separate and their relative motions are highly circumscribed. Thus, through-bond processes are explicitly prevented, as no such bond exists, and although the chromophores are able to move relative to one another in defined ways thanks to the flexibility of the interlocked structure, they are prevented from diffusing apart.

**Fig. 1:**
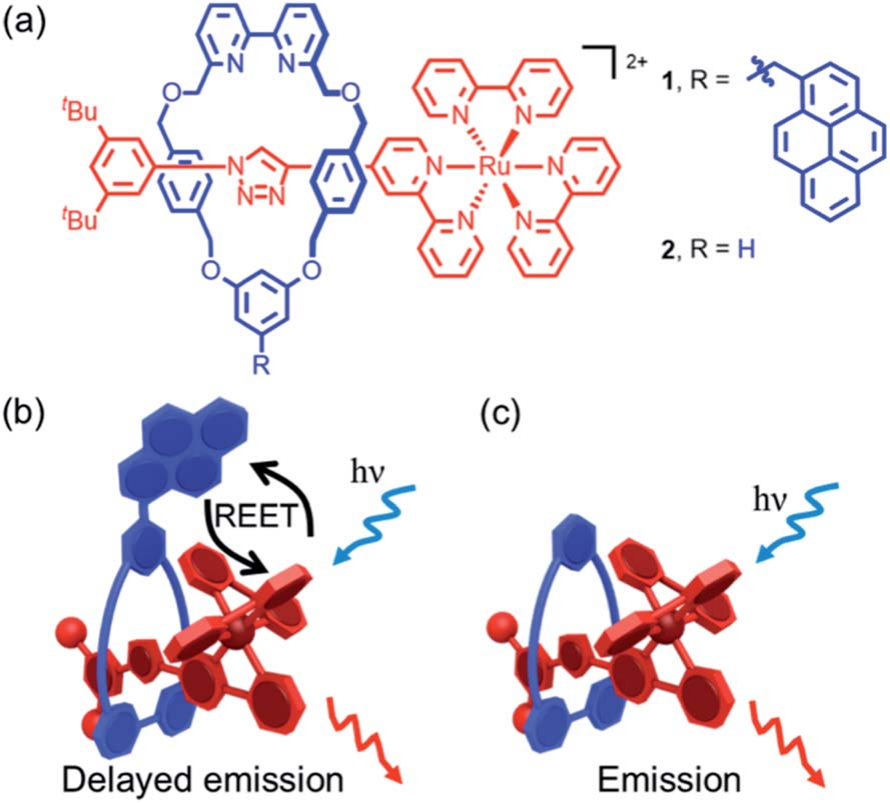
(a) Rotaxanes **1** and **2**. (b) Cartoon of the REET process and consequent delayed luminescence in bichromophoric [2]rotaxane **1**; (c) cartoon of the luminescent process in simple pyrene-free rotaxane **2**.

In the current work, we sought to study reversible electronic energy transfer (REET) between matched chromophores within a rotaxane framework, as represented in Fig. 1. Such REET processes have previously been observed between covalently-linked chromophores^12^ but equivalent processes have not been observed between independent molecules in solution, at least in part due to the strong distance dependence of the REET phenomenon, and triplet–triplet energy transfer in general. The rotaxane scaffold serves as a suitable platform to study this relatively rare photochemical behaviour. Furthermore, we show that binding of a cation into the cavity formed by the mechanical bond modulates the REET process and thus the luminescence response of the system. To our knowledge, this is the first report of a chemical stimulus modulating REET, suggesting a new luminescence lifetime-based reporting mode for the development of molecular chemosensors.

## Results and discussion

The prerequisites for interchromophore REET, following photoexcitation, are that their lowest-lying energy levels are quasi-isoenergetic (within a few hundred cm^−1^) and that the kinetics for energy transfer should be faster than other major deexcitation processes.^12,13^ These conditions can be satisfied using combinations of transition metal complexes (microsecond excited-state lifetimes) and organic chromophores (millisecond excited-state triplet lifetimes), which allows ample time for bidirectional energy transfer. Thus, prototype bichromophoric rotaxane **1** (Fig. 1a) was designed with a pyrene-like chromophore on the ring component and a Ru(bpy)_3_^2+^-based chromophore (bpy = 2,2′-bipyridine) as a stopper of the axle component.^14^ Rotaxane **1** was prepared using an active template^15^ Cu-mediated azide–alkyne cycloaddition (AT-CuAAC)^16^ reaction between the corresponding pyrene-functionalized bipyridine-containing macrocycle (Scheme S1†),^17^ an acetylene functionalized Ru(bpy)_3_^2+^ complex, and an aryl azide (Scheme S2†). Analogue **2**, which lacks the pyrene chromophore, was prepared as a reference compound (Scheme S2†).

The electronic absorption spectrum of **1** (Fig. S1†) contains the distinct absorption bands of both the pyrene and Ru(bpy)_3_^2+^ chromophores, indicating minimal ground-state electronic interaction. The MLCT absorption bands in the visible spectral region in **1** and **2** are identical, as is the red room temperature MLCT-based emission (*λ*_em. max._ = 620 nm, *λ*_ex_ = 450 nm, *Φ* = 0.11 for both) in degassed CH_3_CN (Fig. S2†). A small energy difference (Δ*E* = 515 cm^−1^) between the lowest-lying triplets of the two chromophores in rotaxane **1**, as required for REET, was estimated as the difference between the highest energy MLCT and pyrene features in 77 K phosphorescence spectra (Fig. S3†).

**Fig. 2:**
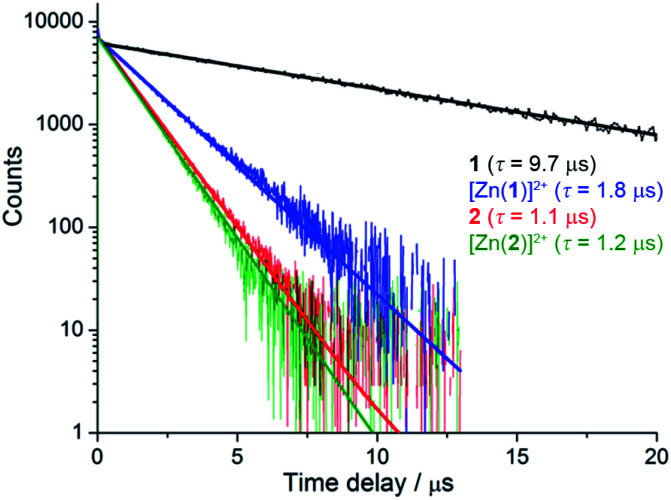
Luminescence decays of rotaxanes **1**, [Zn(**1**)]^2+^, **2** and [Zn(**2**)]^2+^ in degassed acetonitrile (*λ*_ex_ = 450 nm, *λ*_em_ = 620 nm).

Despite similar emission spectra, enhanced O_2_ sensitivity was observed for **1** compared with **2** (*Φ*_deoxy_/*Φ*_oxy_ = 18.3 *vs. Φ*_deoxy_/*Φ*_oxy_ = 5.0). This is consistent with enhanced quenching by dissolved O_2_ in the case of **1** due to a longer excited-state lifetime, as confirmed by luminescence decay values of *τ* = 9.75 μs *vs. τ* = 1.2 μs for **1** and **2**, respectively (Fig. 2). Analysis of luminescence decays on shorter timescales at 300 K (Fig. S4†) revealed an additional short decay component (*τ* ≈ 15 ns) in the case of **1** but not **2**, which corresponds to the time required for energy partitioning between the chromophores to give a thermally-equilibrated excited system, whose deexcitation corresponds to the slow component.

**Fig. 3:**
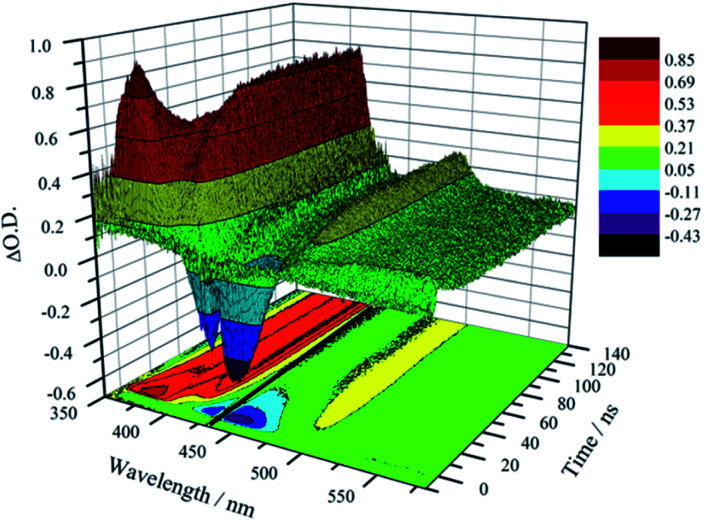
Transient absorption map showing excitation energy redistributing between pyrene and Ru(bpy)_3_^2+^ in **1** in CH_3_CN (*λ*_ex_ = 450 nm) at 300 K. Note: artefact at 442 nm is due to notch filter blocking parasite laser excitation.

Unambiguous evidence for the formation of an excited-state equilibrium as a result of REET between ring and stopper was provided by transient absorption spectroscopy (Fig. 3). Selective excitation in the MLCT band of the Ru(bpy)_3_^2+^ chromophore revealed an initial negative ground state-bleaching band (and positive signature at 370 nm) that evolves synchronously with the growth of a positive band (410 nm) ascribed to the pyrene T_*n*_ ← T_1_ transient with a time constant similar to that of the prompt luminescence decay component (Fig. S5† for kinetic analysis). This corresponds to the real-time observation of energy redistribution between the rotaxane ring and axle chromophores, with a rate that is the sum of forward (*k*_f_) and back (*k*_b_) interchromophore energy transfer rates.^13^

After the initial energy redistribution between ring and axle chromophores, the concomitant decay of the transient signals associated with pyrene triplet and MLCT transient unambiguously confirmed that a dynamic excited-state equilibrium had been established. The dynamic behaviour of excited rotaxane **1**, which is governed by interplay of axle and ring components, was mathematically modelled and described by a set of differential equations (ESI†) and may further be rationalised by the equation below. Here *α* and (1 − *α*) correspond to the relative populations of the excited ring and axle chromophores at equilibrium, respectively. Using this notation, *α*/(1− *α)* equates to the excited-state equilibrium constant, *K*_eq_.
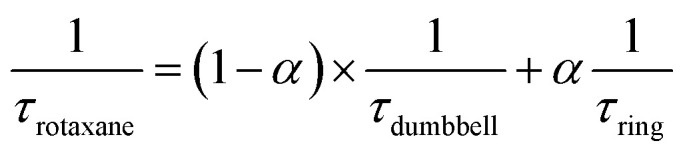


Analysis of variable temperature spectroscopic data (see ESI†) afforded a triplet–triplet energy gap of 475 cm^−1^, similar to the value obtained from the low temperature phosphorescence data (515 cm^−1^) and the population of each excited state at equilibrium as a function of temperature, which was also determined through kinetic analysis. At equilibrium at 298 K, the excitation energy is predominantly localized on the ring pyrene unit (81%) compared with the inorganic stopper (19%), giving a *K*_eq_ of *ca.* 4. Furthermore, the rate of the back transfer (from ring to stopper) and forward (stopper to ring) transfer was calculated (7.07 × 10^7^ s^−1^*vs.* 5.36 × 10^8^ s^−1^, respectively).

Having demonstrated that REET occurs efficiently in rotaxane **1**, we turned our attention to how this process might be modulated using the properties of the interlocked structure. Rotaxanes with cavities similar to those of **1** and **2** have been shown to bind first row transition metal ions in a 1 : 1 stoichiometry in a mechanically chelating fashion involving the bipyridine unit and the triazole.^18^ As the triazole unit of **1** is directly conjugated to the Ru(bpy)_3_^2+^ chromophore, coordination of an ion such as Zn^2+^ is expected to stabilize the MLCT state by rendering the ligand more electron deficient. Even a slight modification of the ^3^MLCT energy level with respect to that of the pyrene, which is largely insensitive to the medium, is expected to perturb and potentially interrupt the REET process with an accompanying effect on luminescence lifetime. Thus, we embarked on a proof-of-principle demonstration of luminescent lifetime-based sensing through analyte-mediated perturbation of REET, an unexplored approach that may have advantages for time-gated detection and sensor concentration-independent measurements.

^1^H-NMR analysis (Fig. S7†) of rotaxane **1** in the absence or presence of Zn^2+^ revealed differences consistent with binding of the metal ion in the rotaxane cavity; several resonances were seen to shift, notably the triazole C–H and the central proton of the resorcinol moiety of the macrocycle. Spectrophotometric titrations of rotaxane **1** with Zn(ClO_4_)_2_·6H_2_O (Fig. S8†) were carried out to further investigate ion binding and obtain binding constants. On adding Zn^2+^, the MLCT absorption bands (400–500 nm) became slightly less intense and broader. Several isosbestic points (477 nm, 374 nm and 325 nm) along with the Job plot (Fig. S9†) are consistent with a 1 : 1 stoichiometry for Zn^2+^ binding by **1**. Unsurprisingly, binding constants of the same order of magnitude were obtained for rotaxanes **1** and **2** (7.14 ± 2.6 × 10^6^ M^−1^ and 1.15 ± 0.2 × 10^6^ M^−1^, respectively), as they integrate structurally similar binding cavities (Fig. S10†).

**Fig. 4:**
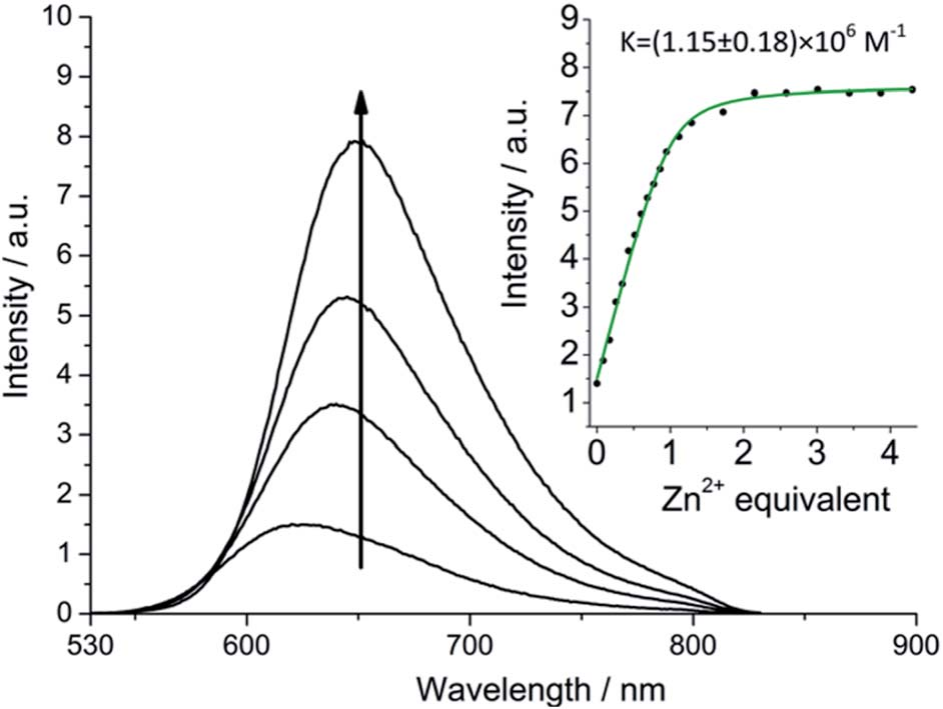
Luminescence spectra (*λ*_ex_ = 475 nm) of **1** on adding Zn(ClO_4_)_2_ in air equilibrated acetonitrile showing ion-regulated oxygen sensitivity (equivalents of Zn^2+^ in order of increasing emission intensity at 650 nm: 0, 0.4, 0.8, 4.5). Inset: changing emission intensity (*λ* = 640 nm) on adding Zn^2+^.

More striking Zn^2+^-induced changes were observed in the luminescence spectra. The luminescence band maximum of **1** (CH_3_CN, *λ*_ex_ = 475 nm, air-equilibrated) shifts from 626 nm to 649 nm (Δ*ω* = 705 cm^−1^) on adding Zn^2+^ and the emission intensity increases (*Φ*([Zn(**1**)]^2+^)/*Φ*(**1**) = 5, Fig. 4). In the case of **2**, Zn^2+^ coordination gives rise to a similar shift in the emission maxima, but with a smaller luminescence enhancement (*Φ*([Zn(**2**)]^2+^)/*Φ*(**2**) = 1.5, Fig. S10†). The enhanced luminescence efficiency of rotaxane **1** in the presence of Zn^2+^ is consistent with an ion-induced disruption of energy shuttling between the axle and macrocycle chromophores as, if this process is rendered less efficient, the lifetime of the emission is shortened and quenching by ambient oxygen is reduced, resulting in luminescence turn “on”. Luminescence decay analysis supports this hypothesis as the delayed luminescence lifetime components of rotaxanes **1** and **2** in degassed solution in the presence of 5 eq. Zn^2+^ are, respectively, 1.8 μs and 1.2 μs *versus* 9.75 μs and 1.1 μs for **1** and **2** alone (Fig. 2).

**Fig. 5:**
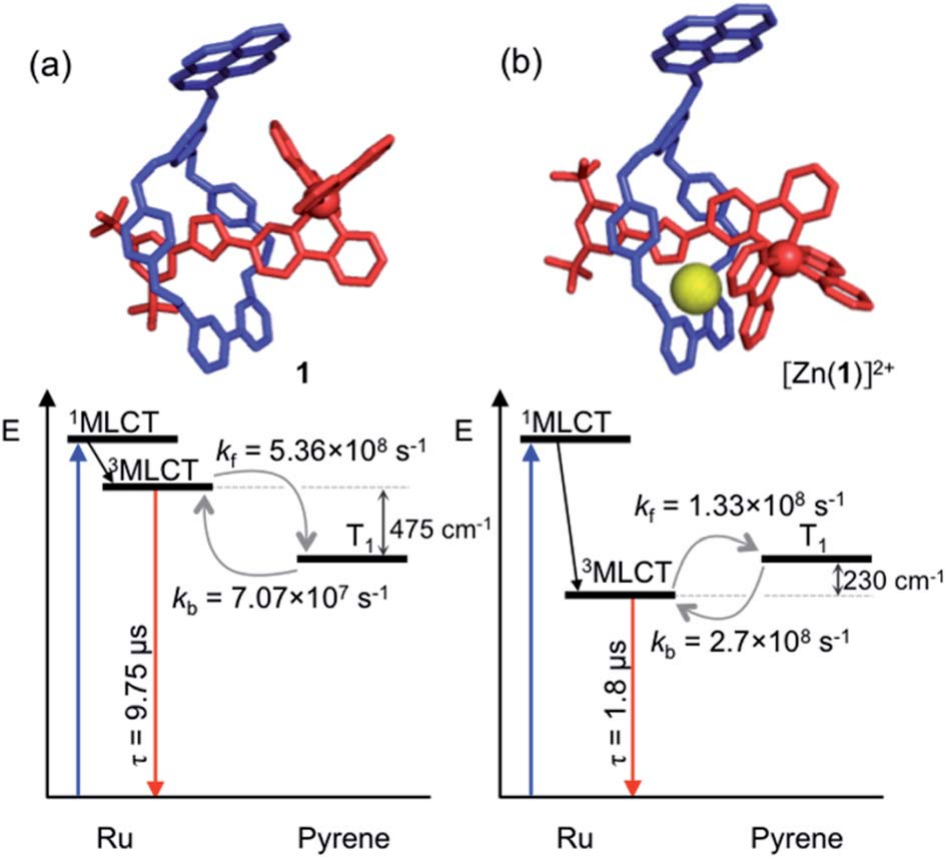
Jablonski–Perrin diagram representing pertinent low lying excited states and electronic energy transfer between ^3^MLCT and triplet pyrene of rotaxanes **1** (a) and [Zn(**1**)]^2+^ (b) at 300 K. Molecular modelling of rotaxane structures used Spartan'18 – MMFF force field.^19^

The transient absorption map of excited [Zn(**1**)]^2+^ shows several qualitatively similar features to that of **1** (see Fig. S11 and kinetic analysis in Fig. S12†). However, after the initial relaxation (*τ* = 13 ns), a larger degree of the excitation energy is localized on the axle chromophore (67% *versus* 19% for **1**). This demonstrates that the excited-state equilibrium is significantly perturbed by ion binding, which is reflected in a luminescence lifetime modulation and increased emission intensity in oxygenated solution because of decreased O_2_ sensitivity. Indeed, binding of Zn^2+^ stabilizes/lowers the ^3^MLCT state energy by *ca.* 700 cm^−1^, while the pyrene triplet states are unaltered, thus decreasing the proportion of energy that is temporarily stored on the pyrene group. Fig. 5 summarises the ensemble of pertinent low-lying states, relative energies and rates of ring-axle energy transfer processes in **1** compared with [Zn(**1**)]^2+^.

Finally, as might be anticipated from the above discussion, the analogous titration of **1** with Zn^2+^ in degassed solution is rather different from that obtained under oxygenated conditions (Fig. S13†). The steady-state emission intensity of **1** varies much less on adding Zn^2+^ (ratio 1 : 1.4) and an isoemissive point (616 nm) is observed. Thus, whereas under oxygenated conditions rotaxane **1** reports the binding of Zn^2+^ in a “switch on” manner, under deoxygenated conditions the rotaxane host facilitates ratiometric detection of Zn^2+^ by monitoring unchanging emission intensity (616 nm) *versus* significant MLCT changes at, for example, 645 nm, making Zn^2+^ detection largely rotaxane concentration independent.

## Conclusions

We have reported the first example of reversible electronic energy transfer between chromophores that are not covalently linked by using the mechanical bond as a platform. Integrating energetically and kinetically matched chromophores onto the ring and axle units of a rotaxane is shown to offer a design principle to enable fast reversible intercomponent energy exchange to take place resulting in delayed luminescence and long luminescence lifetimes. The reversibility of the energy transfer process is shown to be highly efficient as identical luminescence quantum yields are measured in the bichromophoric rotaxane and pyrene-free variant, indicating no significant additional energy loss pathway in the current case. Furthermore, the high sensitivity of this energy shuttling to perturbation of the relative energies of the excited states involved in the REET process was used to develop a simple chemosensor with the potential for ratiometric detection of metal ions, an unexplored sensing mechanism. This rotaxane platform could further be adapted to integrate alternative kinetically and energetically matched dye pairs exhibiting REET, to regulate excitation and emission wavelength, luminescence lifetime range and modulate oxygen sensitivity. We are now exploring coupled ring and energy shuttling as a method for developing new approaches to lifetime-based chemosensing and photosensitizer development.

## Data availability

Principle data and characterisation is available in ESI.

## Author contributions

All authors contributed to manuscript preparation. Additionally:- SY: Synthesis, characterization, titrations; AK: Steady-state & time-resolved spectroscopy; JEML: Synthesis, analysis and supervision. VM-C: Experimental revisions & molecular modelling; GJ: Fast spectroscopy & data analysis; J-LP: Supervision & editing; SMG: Molecule design, analysis and supervision. NMcC: Concepts, photoactive molecule designs, supervision and data analysis.

## Conflicts of interest

There are no conflicts to declare.

## Supplementary Material

SC-012-D1SC02225C-s001
