# Supplementary material for: Damming an electronic energy reservoir: ion-regulated electronic energy shuttling in a [2]rotaxane
Source: Chem Sci. 2021 Jun 4;12(26):9196–200. doi: 10.1039/d1sc02225c (PMC8261707; doi:10.1039/d1sc02225c)
Supplement: SC-012-D1SC02225C-s001 [file SC-012-D1SC02225C-s001.pdf]

Electronic Supplementary Material (ESI) for Chemical Science.
This journal is © The Royal Society of Chemistry 2021

Supporting Information

Damming an electronic energy reservoir: ion-regulated electronic energy shuttling in a [2]rotaxane

Shilin Yu, Arkady Kupryakov, James E. M. Lewis, Vicente Martí-Centelles, Stephen M. Goldup,* Jean-Luc Pozzo, Gediminas Jonusauskas and Nathan D. McClenaghan*

Email: nathan.mcclenaghan@u-bordeaux.fr; s.goldup@soton.ac.uk

1. Materials and Methods S2
2. Synthetic Procedures S4
3. Optical Spectroscopy : Electronic absorption & Luminescence S13
4. Detailed Photophysical Interpretation and Kinetic Analysis S16
5. Zinc-induced Spectral & Dynamics Changes S19
6. NMR Spectra S23
7. References S43

1. Materials and Methods

All reactions were carried out under an atmosphere of nitrogen using oven-dried glassware unless otherwise stated. DIPEA refers to N,N-diisopropylethylamine. 1-Azido-3,5-di-*tert*-butylbenzene **5**[S1], [Ru(bipy)$_2$(bipy-CCH)](PF$_6$)$_2$, **6**[S2] and 6-bromo-2-pyridinemethanol **7**[S3] were prepared according to previously published procedures. Reagent grade tetrahydrofuran (THF) was distilled under argon over sodium benzophenone ketyl. CH_2Cl_2 was distilled over CaH_2 under argon. All other reagents were purchased from commercial suppliers and used without further purification. Flash column chromatography was carried out using silica gel (230-400 mesh). Thin layer chromatography (TLC) was carried out on plates coated with silica gel 40 F254 purchased from Aldrich.

^{1}H NMR and ^{13}C NMR spectra were recorded at 300 MHz and 600 MHz, on a Bruker Avance 300 spectrometer or an Avance III 600 spectrometer. Proton chemical shifts (δ) are reported in ppm downfield from tetramethylsilane (TMS). Mass spectrometry was performed by the CESAMO analytical centre (University of Bordeaux, France) on a QStar Elite mass spectrometer (Applied Biosystems). The instrument is equipped with an ESI source and spectra were recorded in the positive mode. The electrospray needle was maintained at 5000 V and operated at room temperature. Samples were introduced by injection through a 20 µL sample loop into a 4500 µL/min flow of methanol from the LC pump.

Electronic absorption spectra were recorded on a spectrophotometer Cary 5G UV-Vis-NIR (Varian) using 10 mm and 1 mm synthetic quartz (Suprasil) quartz cells. Luminescence (fluorescence and phosphorescence at temperatures 77 K and 295 K) spectra were recorded on a Fluorolog-3 (Jobin Yvon) spectrophotometer with iHR-320 spectrograph and photomultipliers from Hamamatsu Photonics: R928 and R2658. Fluorescence quantum yield studies were performed using optically dilute samples on comparing emission intensity with a standard of known quantum yield, $[Ru(bpy)_3]^{2+}$ in non-degassed H_2O ($\Phi_{em} = 0.028$).[S4] All spectra were recorded as an average of 5 scans and corrected. The transient absorption / time-resolved luminescence set-up was built as follows: a frequency tripled Nd:YAG amplified laser system (30 ps, 30 mJ @1064 nm, 20 Hz, Ekspla model PL 2143) output was used to pump an optical parametric generator (Ekspla model PG 401) producing tunable excitation pulses in the range 410 – 2300 nm. The residual fundamental laser radiation was focused in a high pressure Xe filled breakdown cell where a white light pulse for sample probing was produced. All light signals were analyzed by a spectrograph (Princeton Instruments Acton model

SP2300) coupled with a high dynamic range streak camera (Hamamatsu C7700, 1 ns - 1 ms). Accumulated sequences (sample emission, probe without and with excitation) of pulses were recorded and treated by HPDTA (Hamamatsu) software to produce two-dimensional maps (wavelength vs delay) of transient absorption intensity in the range 300 – 800 nm. Typical measurement error was better than 10^{-3} O. D. Data were analysed using home-made software developed in LabVIEW 2014 system-design platform and development environment. The trust-region dogleg algorithm (supported by LabVIEW 2014) was applied to determine the set of parameters that best fit the set of input data. The trust-region dogleg algorithm was used instead of Levenberg-Marquardt algorithm, the latter being less stable in most cases during optimization process, because trust region methods are robust, and can be applied to ill-conditioned problems.

2. Synthetic Procedures

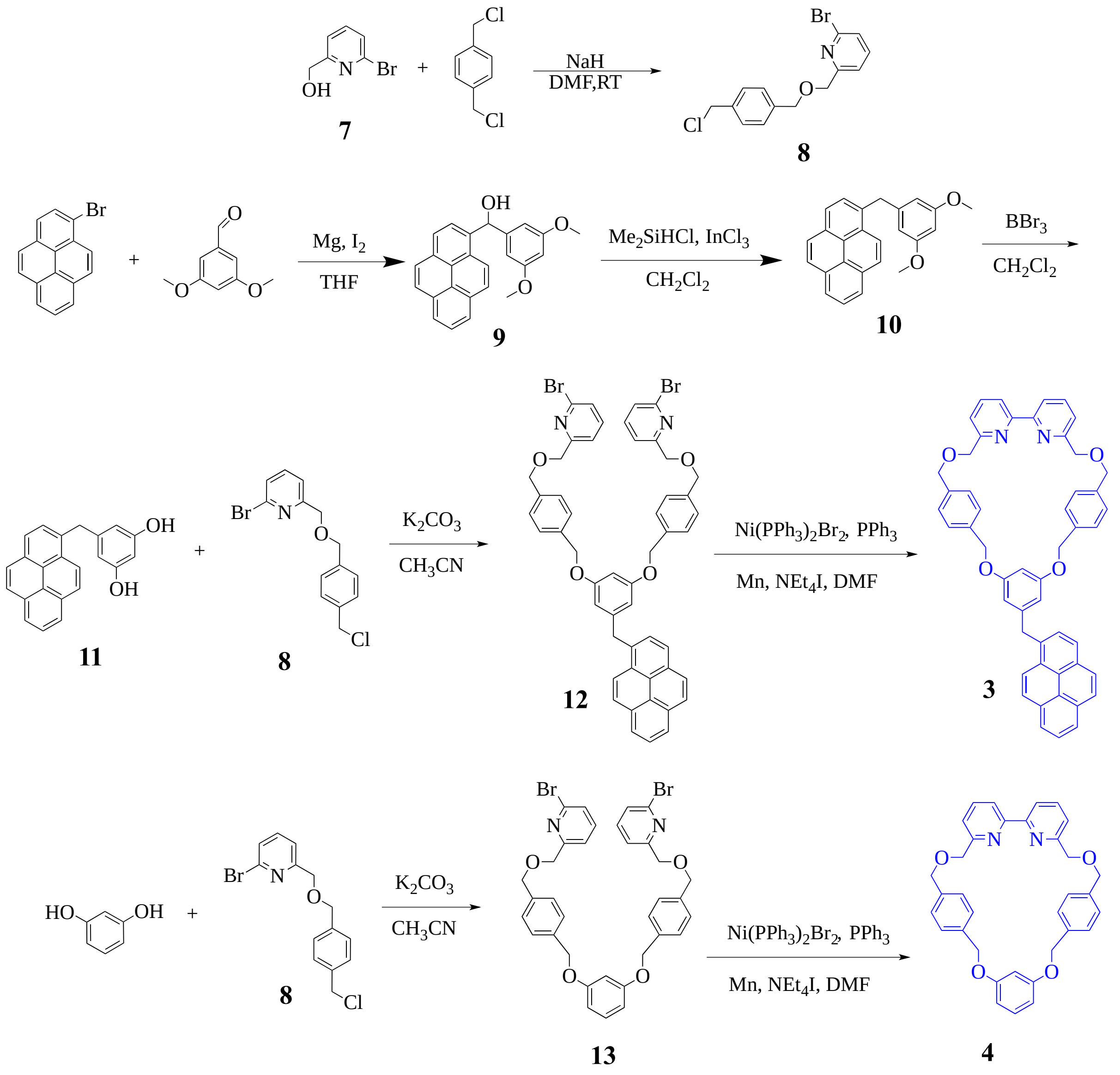

Scheme S1: Synthesis of macrocycles **3** and **4**.

Synthesis of 8

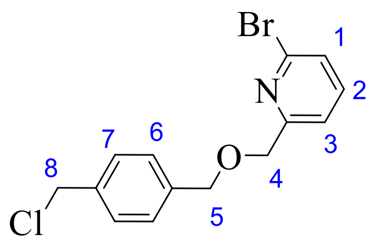

6-Bromo-2-pyridinemethanol **7** (1.00 g, 5.4 mmol, 1 eq) in DMF (40 mL) was added dropwise into a solution of 4-(chloromethyl)benzylchloride (2.79 g, 16.1 mmol, 3 eq) and NaH (0.26 g, 11.1 mmol, 2 eq) in DMF (30 mL) and the mixture was stirred overnight at room temperature. The mixture was washed with a saturated aqueous solution of NH_4Cl (80 mL) and extracted with dichloromethane (2 × 75 mL). The combined organic layers were dried over $MgSO_4$ and the solvent was removed *in vacuo*. The crude product was purified by silica gel column chromatography (cyclohexane/AcOEt 90:10, v/v), affording **8** as a yellow solid (0.94 g, yield = 54%). **^{1}H NMR** (300 MHz, $CDCl_3$): δ 7.56 (t, *J* = 7.2 Hz, 1H, H_2), 7.48 - 7.44 (m, 1H, H_1), 7.41 - 7.34 (m, 5H, H_3, H_6, H_7), 4.66 (s, 2H, H_4), 4.64 (s, 2H, H_5), 4.59 (s, 2H, H_8). **^{13}C NMR** (75 MHz, $CDCl_3$): δ 160.2, 141.3, 139.0, 138.0, 137.1, 128.8, 128.1, 126.7, 120.0, 72.6, 72.4, 46.0. **HRMS (ESI+):** calcd for $C_{14}H_{13}NaBrClNO$ *m/z* = 347.9761, found *m/z* = 347.9767.

Synthesis of 9

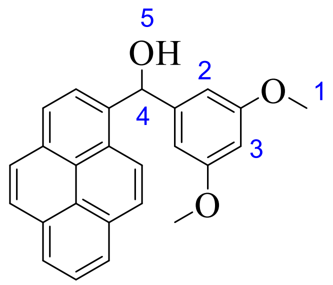

A solution of n-BuLi in hexane (6.7 mL of 2.4 M) was added dropwise to a solution of 1-bromopyrene (3.0 g, 10.7 mmol, 1 eq) in THF (10 mL) under Ar atmosphere at -78 °C. After 30 min. 3,5-dimethoxybenzaldehyde (1.8 g, 10.7 mmol, 1 eq) was slowly added to the solution. The reaction mixture was stirred for 2 h at room temperature and then quenched by the addition of an aqueous solution of NH_4Cl. The organic layer was extracted with ether and washed with water and brine, dried over Na_2SO_4 and the solvent was removed *in vacuo*. The crude product was purified by silica gel column chromatography (cyclohexane/AcOEt 3:1, v/v), affording **9** as a white solid (3.3 g, yield = 84%). **^{1}H NMR** (300 MHz, $CDCl_3$): δ 8.41 (d, *J* = 12 Hz, 1H, *Pyrene*), 8.26 - 8.18 (m, 4H, *Pyrene*), 8.15 - 8.02 (m, 4H, *Pyrene*), 6.86 (d, *J* =3.9 Hz, 1H, H_4), 6.67 (d, *J* = 1.8 Hz, 2H, H_2), 6.42 (t, *J* = 2.4 Hz, 1H, H_3), 3.77 (s, 6H, H_1), 2.50 (d, *J* = 3.9 Hz, 1H, H_5). **^{13}C NMR** (75 MHz, $CDCl_3$): δ 160.9, 146.2, 136.4, 131.3, 131.0, 130.6, 128.2, 127.8, 127.5, 127.43, 127.42, 126.0, 125.3, 125.2, 125.0, 124.9, 124.8, 123.0, 105.1, 99.4, 73.4, 55.3. **HRMS (ESI+):** calcd for $C_{25}H_{20}O_3Na$ *m/z* = 391.1304, found *m/z* = 391.1310.

Synthesis of 10

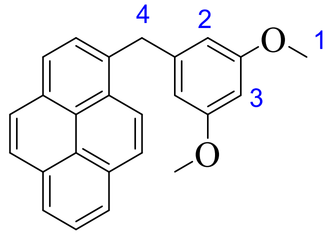

To a solution of compound **9** (2.50 g, 6.8 mmol, 1 eq) and $InCl_3$ (75 g, 0.05 eq) in 30 mL of CH_2Cl_2 was added Me_2SiHCl (1.53 g, 16.2 mmol, 2.4 eq). The solution was stirred for 6 h at room temperature. The reaction mixture was diluted with CH_2Cl_2 and washed with water and brine, dried over Na_2SO_4, and the solvent was removed *in vacuo*. The residue was purified by a silica gel column chromatography (cyclohexane/AcOEt 95:5, v/v) to give **10** as a white solid (2.06 g, yield = 86%). **^{1}H NMR** (300 MHz, $CDCl_3$): δ 8.25 (d, *J* = 12 Hz, 1H, *Pyrene*), 8.19 - 8.12 (m, 3H, *Pyrene*), 8.08 - 7.96 (m, 4H, *Pyrene*), 7.88 (d, *J* = 7.8 Hz, 1H, *Pyrene*), 6.38 (d, *J* = 2.1 Hz, 2H, H_2), 6.30 (t, *J* = 2.4 Hz, 1H, H_3), 4.68 (s, 2H, H_4), 3.69(s, 6H, H_1). **^{13}C NMR** (75 MHz, $CDCl_3$): δ 160.9, 143.7, 134.2, 131.4, 130.9, 130.3, 129.3, 128.2, 127.55, 127.52, 126.9, 125.9, 125.2, 125.0, 124.92, 124.91, 127.89, 123.7, 107.1, 97.9, 55.2, 39.6. **HRMS (ESI+):** calcd for $C_{25}H_{20}O_2Na$ *m/z* = 375.1355, found *m/z* = 375.1356.

Synthesis of 11

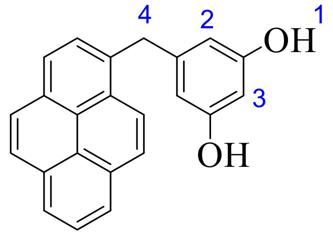

Boron tribromide (0.50 mL, 4.9 mmol, 2.7 eq) was added slowly to a solution of **10** (653 mg, 1.8 mmol, 1 eq) in dichloromethane (40 mL) at 0°C. The mixture was stirred 2 h at 0°C, and stirred overnight at room temperature. Methanol (6 mL) followed by water (50 mL) was added slowly to the mixture and stirred for a further 2h at room temperature. The mixture was washed with a saturated aqueous solution of $NaHCO_3$ (100 mL) and extracted with dichloromethane (2 × 75 mL). The organic layer was dried over $MgSO_4$ and solvent was removed *in vacuo*. The crude product was purified by silica gel column chromatography (cyclohexane/AcOEt 6:4, v/v), affording of **11** as a white solid (515 mg, yield = 86%). **^{1}H NMR** (300 MHz, $CDCl_3$): δ 8.22 - 8.13 (m, 4 H, *Pyrene*), 8.10 - 7.96 (m, 3 H, *Pyrene*), 8.00 (t, *J* = 7.6 Hz, 1H, *Pyrene*), 7.88 (d, *J* = 7.8 Hz, 1H, *Pyrene*), 6.23 (m, 2 H, H_2), 6.17 (m, 1 H, H_3), 4.62 (s, 2H, H_4), 4.60 (br s, 2 H, H_1). **^{13}C NMR** (75 MHz, Tetrahydrofuran-d_8): δ 158.9, 143.1, 135.0, 131.5, 131.0, 130.2, 129.4, 128.4, 127.3, 127.0, 126.5, 125.7, 125.0, 124.8, 124.7, 124.6, 124.1, 106.7, 100.2, 38.9 (20 signals found, 22 signals expected, 2 signals missing/coincidental). **HRMS (ESI+):** calcd for $C_{23}H_{17}O_2$ *m/z* = 325.1223, found *m/z* = 325.1228.

Synthesis of pyrene-macrocycle precursor 12

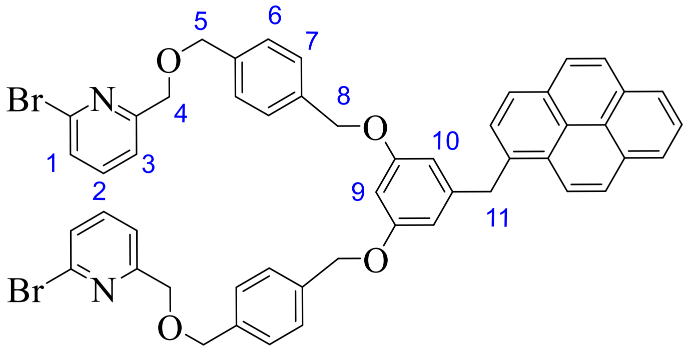

Compound **8** (813 mg, 2.50 mmol, 2 eq) was added to a solution of **11** (405 mg, 1.25 mmol, 1 eq) and K_2CO_3 (1.04 g, 7.50 mmol, 6 eq) in acetonitrile (45 mL). The reaction mixture was heated to reflux overnight. The white suspension was filtered, washed with acetonitrile (40 mL), and the solvent was removed *in vacuo*. The crude product was purified by silica gel column chromatography (cyclohexane/AcOEt 3:1, v/v), affording **12** as a yellow solid (721 mg, yield = 64%). **^{1}H NMR** (300 MHz, $CDCl_3$): δ 8.27 – 8.14 (m, 4H, *Pyrene*), 8.21 – 8.11 (m, 4H, *Pyrene*), 7.90 (d, *J* = 6.9 Hz, 1H, *Pyrene*), 7.57 (t, *J* = 7.6 Hz, 2H, H_2), 7.47 (dd, *J* = 7.6, 0.8 Hz, 2H, H_3), 7.40 (dd, *J* = 7.4, 0.9 Hz, 2H, H_1), 7.20 – 7.13 (m, 8H, H_6, H_7), 6.35(s, 3H, H_9, H_{10}), 4.82 (s, 4H, H_8), 4.55 (s, 2H, H_{11}), 4.53 (s, 4H, H_4), 4.45 (s, 4H, H_5). **^{13}C NMR** (75 MHz, $CDCl_3$): δ 160.3, 160.0, 143.7, 141.3, 139.0, 137.4, 136.5, 134.0, 131.4, 130.9, 129.3, 128.3, 127.9, 127.6, 127.5, 126.9, 126.6, 125.9, 125.2, 125.1, 124.92, 124.90, 124.88, 123.8, 120.0, 108.1, 99.9, 72.7, 72.3, 69.7, 39.5 (31 signals found, 33 signals expected, 2 signals missing/coincidental). **HRMS (ESI+):** calcd for $C_{51}H_{40}NaBr_2N_2O_4$ *m/z* = 925.1247, found *m/z* = 925.1250.

Synthesis of pyrene-macrocycle 3

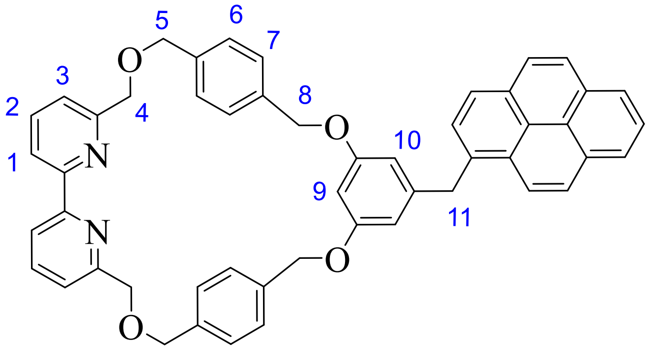

Dibromobis(triphenylphosphine)nickel(II) (421 mg, 0.566 mmol, 1 eq), triphenylphosphine (297 mg, 1.132 mmol, 2 eq), manganese(0) (312 mg, 5.66 mmol, 10 eq) and tetraethylammonium iodide (146 mg, 0.566 mmol, 1 eq) were dissolved in DMF (5 mL) and sonicated for 10 min followed by heating for 1 h at 50°C. **12** (511 mg, 0.566 mmol, 1 eq) was dissolved in DMF (5 mL) and added to the reaction mixture via a syringe pump over 4 h at 50 °C. The mixture was then heated for a further 1 h. A solution of EDTA/NH_4OH(aq) (100 mL) was added and the mixture was stirred at room temperature for 4 min.

The aqueous layer was extracted with dichloromethane (2 × 15 mL), organic layers were combined, washed with water (50 mL), brine (50 mL), and solvent was removed *in vacuo*. The crude product was purified by silica gel flash column chromatography with using a gradient of 0 – 100% Et_2O and petroleum ether, pyrene-macrocycle **3** as a white solid (295 mg, yield = 70%). **^{1}H NMR** (300 MHz, $CDCl_3$): δ 8.25 (d, *J* = 9.3 Hz, 1H, *Pyrene*), 8.17 (dd, *J* = 9.8, 7.6 Hz, 3H, *Pyrene*), 8.09 – 7.96 (m, 4H, *Pyrene*), 7.89 (d, *J* = 7.8 Hz, 1H, *Pyrene*), 7.82 (dd, *J* = 7.8, 1.0 Hz, 2H, H_1), 7.54 (t, *J* = 7.8 Hz, 2H, H_2), 7.18 - 7.06 (m, 10H, H_3, H_6, H_7), 6.43 (d, *J* = 2.1 Hz, 2H, H_{10}), 6.12 (t, *J* = 2.1 Hz, 1H, H_9), 4.89 (s, 4H, H_8), 4.66 (s, 2H, H_{11}), 4.64 (s, 4H, H_4), 4.59 (s, 4H, H_5). **^{13}C NMR** (75 MHz, $CDCl_3$): δ 160.0, 158.3, 155.6, 143.6, 137.6, 137.3, 136.6, 134.2, 131.5, 131.0, 130.4, 129.4, 128.7, 128.5, 127.7, 127.6, 127.05, 127.03, 126.0, 125.3, 125.2, 125.04, 125.02, 125.01, 123.9, 121.7, 120.4, 108.1, 100.4, 72.9, 72.7, 69.8, 39.6. **HRMS (ESI+):** calcd for $C_{51}H_{40}N_2O_4Na$ *m/z* = 767.2880, found *m/z* = 767.2881.

Synthesis of macrocycle precursor 13

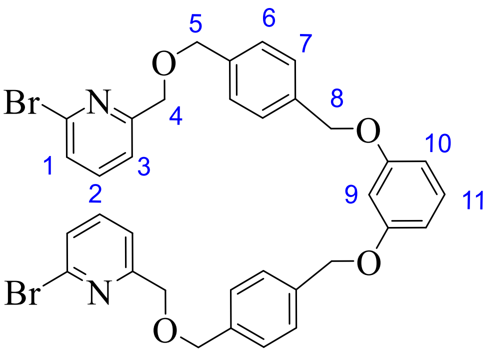

Compound **8** (591 mg, 1.82 mmol, 2 eq) was added to a solution of resorcinol (100 mg, 0.91 mmol, 1 eq) and K_2CO_3 (760 mg, 5.45 mmol, 6 eq) in acetonitrile (45 mL) and the mixture was heated at reflux overnight. The white suspension was filtered and washed with acetonitrile (40 mL) and the solvent was removed *in vacuo*. The crude product was purified by silica gel column chromatography (cyclohexane/AcOEt 3:1, v/v), affording **13** as a yellow solid (500 mg, yield = 80%). **^{1}H NMR** (300 MHz, $CDCl_3$): δ 7.56 (t, *J* = 7.6 Hz, 1H, H_2), 7.50 – 7.34 (m, 12H, H_1, H_3, H_6, H_7), 7.18 (t, *J* = 8.1 Hz, 1H, H_{11}), 6.64 – 6.56 (m, 3H, H_9, H_{10}), 5.04 (s, 4H, H_8), 4.66 (s, 4H, H_4), 4.65 (s, 4H, H_5). **^{13}C NMR** (75 MHz, $CDCl_3$): δ 160.4, 160.1, 141.4, 139.1, 137.6, 136.7, 130.1, 128.2, 127.8, 126.7, 120.1, 107.5, 102.4, 72.9, 72.4, 69.9. **HRMS (ESI+):** calcd for $C_{34}H_{30}Br_2N_2O_4$ *m/z* = 688.0572, found *m/z* = 688.0580.

Synthesis of macrocycle 4

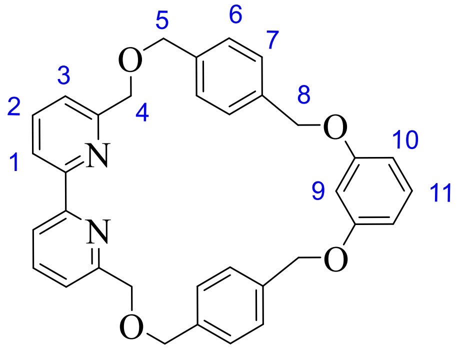

Dibromobis(triphenylphosphine)nickel(II) (507 mg, 0.682 mmol, 1 eq), triphenylphosphine (358 mg, 1.364 mmol, 2 eq), manganese(0) (375 mg, 6.816 mmol, 10 eq) and tetraethylammonium iodide (176 mg, 0.682 mmol, 1 eq) were dissolved in DMF (5 mL) and sonicated for 10 min and heated at 50°C for 1 h. Macrocycle precursor **13** (469 mg, 0.682 mmol, 1 eq) was dissolved in DMF (6.8 mL) and added to the reaction mixture via a syringe pump over 4 h at 50°C. When the addition was finished, the mixture maintained at 50 °C for 1 h, then a solution of EDTA/NH_4OH(aq) (100 mL) was added and the mixture was stirred at room temperature for 4 min. The aqueous layer was extracted with dichloromethane (2 × 20 mL), the combined organic layers were washed with water (50 mL) and brine (50 mL) and the solvent was removed *in vacuo*. The crude product was purified by silica gel flash column chromatography (petroleum ether with a Et_2O gradient from 0 to 100%) to obtain macrocycle **4** as a white solid (253 mg, yield = 70%). **^{1}H NMR** (300 MHz, CD_3CN): δ 8.00 (dd, J = 0.8, 6.1 Hz, 2H, H_1), 7.73 (t, J = 7.7 Hz, 2H, H_2), 7.32 (dd, J = 0.8, 7.8 Hz, 2H, H_3), 7.25 – 7.17 (m, 8H, H_6 and H_7), 7.14 (t, J = 8.3 Hz, 1H, H_{11}), 6.53 (dd, J = 2.4, 8.2 Hz, 2H, H_{10}), 6.22 (t, J = 2.4 Hz, 1H, H_9), 5.01 (s, 4H, H_8), 4.67 (s, 4H, H_4), 4.64 (s, 4H, H_5). **^{13}C NMR** (75 MHz, $CDCl_3$): δ 160.0, 158.6, 155.8, 137.7, 137.3, 136.8, 129.9, 128.8, 127.1, 121.0, 120.4, 107.6, 102.8, 73.0, 72.8, 69.9. **HRMS (ESI+):** calcd for $C_{34}H_{30}N_2O_4$ m/z = 530.2206, found m/z = 530.2200.

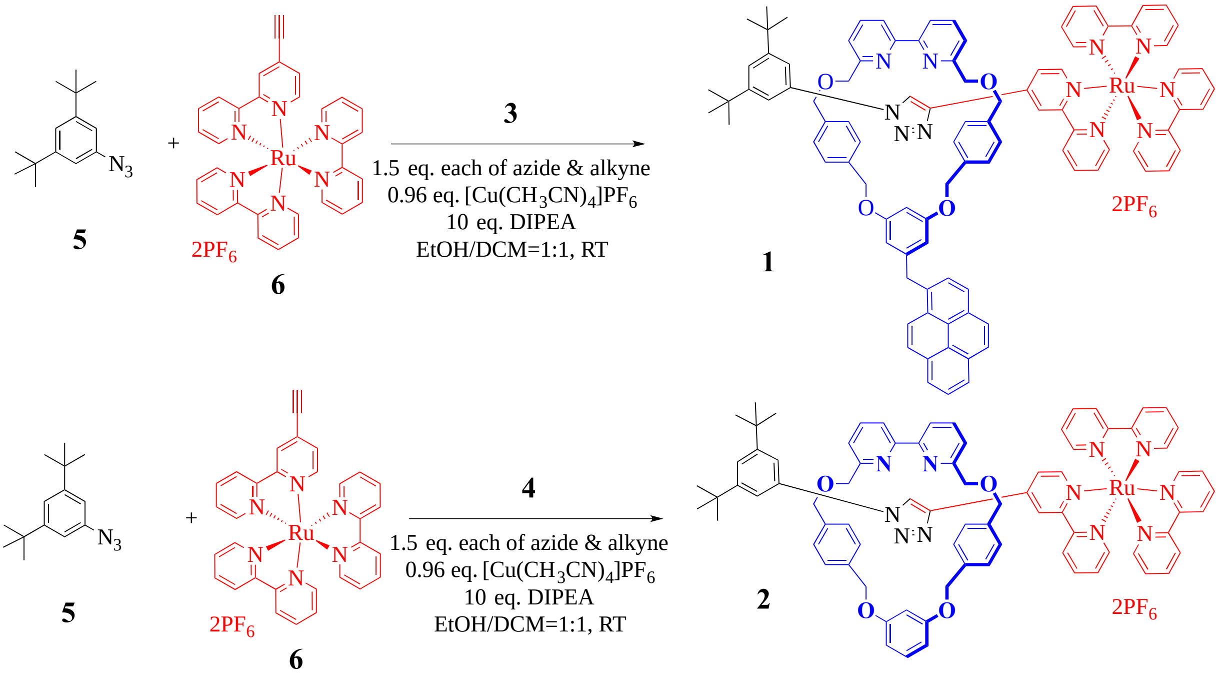

Scheme S2: Synthesis of rotaxanes **1** and **2**.

Rotaxane 1

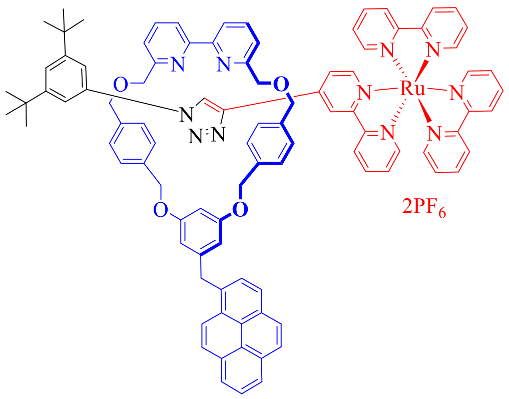

A CEM vial was charged with macrocycle **3** (22.4 mg, 0.030 mmol, 1 eq), $[Cu(MeCN)_4](PF_6)$ (10.7 mg, 0.029 mmol, 0.96 eq), azide **5** (6.9 mg, 0.0627 mmol, 1.5 eq) and alkyne **6** (40.0 mg, 0.045 mmol, 1.5 eq) in 1:1 EtOH/DCM (3.0 mL). Then DIPEA (52 μL, 10 eq) was added to the reaction mixture. The deep red solution was stirred overnight at room temperature. The reaction mixture was then diluted with DCM (40 mL) and washed EDTA/NH_4OH(aq) (50 mL). The aqueous layer was extracted with dichloromethane (2 × 40 mL), the combined organic layers was washed with water (50 mL) and brine (50 mL), dried over $MgSO_4$ and solvent was removed *in vacuo*. The crude solid was purified by silica gel column chromatography (acetonitrile/water/KNO_3 (sat. aq.) 90:9.8:0.2, v/v/v) and gel permeation chromatography (GPC). After solvent removal the red solid was dissolved in DCM (5 mL), and washed with a sat. NH_4PF_6 aqueous solution, dried over Na_2SO_4 and solvent removed *in vacuo* to obtain **1** as a red solid (33.5 mg, yield = 60%). **^{1}H NMR** (300 MHz, CD_3CN): δ 9.90 (s, 1H), 8.52 (d, *J* = 1.6 Hz, 1H), 8.46 (dd, *J* = 8.0, 3.3 Hz, 2H), 8.40 (d, *J* = 9.3 Hz, 1H), 8.29 – 8.17 (m, 4H), 8.17 – 8.10 (m, 4H),

8.08 – 8.00 (m, 4H), 7.99 – 7.85 (m, 2H), 7.79 – 7.46 (m, 15H), 7.44 – 7.28 (m, 5H), 7.15 – 7.07 (m, 1H), 6.96 – 6.84 (m, 4H), 6.71 (d, *J* = 5.9 Hz, 1H), 6.64 – 6.61 (m, 1H), 6.53 (d, *J* = 8.2 Hz, 2H), 6.44 – 6.40 (m, 2H), 6.21 (d, *J* = 8.0 Hz, 2H), 5.96 (t, *J* = 2.2 Hz, 1H), 4.92 (d, *J* = 13.1 Hz, 1H), 4.79 (d, *J* = 13.1 Hz, 1H), 4.68 – 4.61 (m, 3H), 4.53 – 4.19 (m, 7H), 4.10 (d, *J* = 12.5 Hz, 1H), 3.98 (d, *J* = 12.5 Hz, 1H), 1.27 (s, 18H). **^{13}C NMR** (150 MHz, CD_3CN): δ 160.80, 160.72, 159.41, 158.81, 158.17, 158.03, 157.75, 157.69, 157.65, 157.57, 157.33, 156.13, 154.00, 152.49, 152.47, 152.40, 152.24, 151.51, 150.47, 145.16, 143.85, 140.26, 139.10, 138.90, 138.76, 138.72, 138.68, 138.44, 138.30, 138.25, 138.10, 137.69, 137.48, 137.33, 135.86, 132.31, 131.72, 131.18, 129.86, 129.43, 129.39, 128.81, 128.77, 128.63, 128.51, 128.49, 128.41, 128.39, 128.28, 128.06, 127.91, 127.21, 127.16, 126.18, 126.07, 125.94, 125.85, 125.79, 125.47, 125.24, 125.17, 124.93, 124.78, 124.23, 123.92, 123.49, 123.13, 122.72, 122.34, 122.21, 115.13, 108.65, 108.28, 102.21, 73.79, 73.76, 73.65, 72.79, 70.55, 70.02, 39.70, 35.77, 31.35. **HRMS (ESI+):** calcd for *m/z* = 784.7909 ($[M - 2PF_6]^{2+}$), found *m/z* = 784.7934.

Rotaxane 2

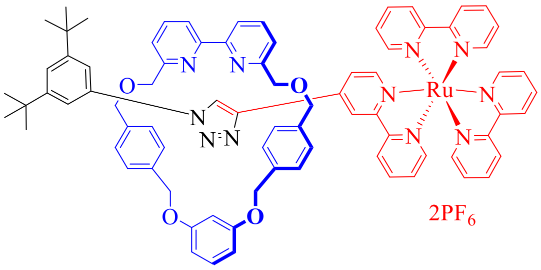

A CEM vial was charged with macrocycle **4** (12.0 mg, 0.023 mmol, 1 eq), $[Cu(MeCN)_4](PF_6)$ (8.1 mg, 0.022 mmol, 0.96 eq), azide **5** (7.8 mg, 0.034 mmol, 1.5 eq) and alkyne **6** (30.0 mg, 0.034 mmol, 1.5 eq) in 1:1 EtOH/DCM (2.3 mL). Then DIPEA (39 μL, 10 eq) was added to the reaction mixture. The deep red solution was stirred overnight at room temperature. The reaction mixture was then diluted with DCM (40 mL) and washed with EDTA/NH_4OH(aq) (50 mL). The aqueous layer was extracted with dichloromethane (2 × 40 mL), the combined organic layers were washed with water (50 mL), brine (50 mL), dried over $MgSO_4$ and solvent was removed *in vacuo*. The crude solid was purified by silica gel column chromatography (acetonitrile/water /KNO_3 (sat. aq.) 90:9.8:0.2, v/v/v) and gel permeation chromatography (GPC). After solvent removal the red solid was dissolved in DCM (5 mL), and washed with sat. NH_4PF_6 aqueous solution, dried over Na_2SO_4 and solvent was removed *in vacuo* to obtain **2** as a red solid (29.7 mg, yield = 80%). **^{1}H NMR** (300 MHz, CD_3CN): ^{1}H NMR (300 MHz, CD_3CN) δ 9.93 (s, 1H), 8.70 – 8.33 (m, 6H), 8.25 – 7.98 (m, 5H), 7.85 – 7.67 (m, 4H), 7.67 – 7.54 (m, 8H), 7.53 – 7.35 (m, 6H), 7.28 – 7.21 (m, 1H), 7.15 (t, *J* = 8.3 Hz, 1H), 7.07 (d, *J* = 8.1 Hz, 2H), 6.92 (d, *J* = 8.2 Hz, 2H), 6.69 – 6.51 (m, 4H), 6.48 – 6.34 (m, 4H), 6.25 (t, *J* = 2.4 Hz, 1H), 5.09 (d, *J* = 13.6 Hz, 1H), 4.97 (d, *J* = 13.5 Hz, 1H), 4.80 (d, *J* = 13.2 Hz, 1H), 4.53 – 4.40 (m, 2H), 4.38 – 4.20 (m, 5H), 4.10 (d, *J* = 12.8 Hz, 1H), 3.99 (d, *J* = 12.8 Hz, 1H), 1.32 – 1.24 (m, 18H). **^{13}C NMR** (150 MHz, CD_3CN): δ 160.78, 160.72, 159.63, 159.02, 158.17, 157.94, 157.82, 157.78, 157.69, 157.35, 156.29, 154.08, 152.63, 152.60, 152.44, 151.55, 150.20, 143.97, 140.32, 139.28, 139.04, 139.01, 138.78, 138.69, 138.60,

138.45, 138.40, 138.30, 137.80, 137.59, 137.49, 131.40, 129.46, 128.92, 128.77, 128.63, 128.52, 128.41, 128.19, 128.10, 127.29, 125.96, 125.57, 125.38, 125.33, 125.22, 124.40, 123.95, 123.53, 123.37, 123.01, 122.86, 122.47, 122.33, 115.19, 108.55, 107.65, 104.03, 73.83, 73.74, 72.86, 70.37, 70.00, 55.32, 35.87, 31.46. **HRMS (ESI+):** calcd for *m/z* = 1349.5080 ($[M - 2PF_6]^{2+}$), found *m/z* = 1349.5105.

3. Optical Spectroscopy : Electronic absorption & Luminescence

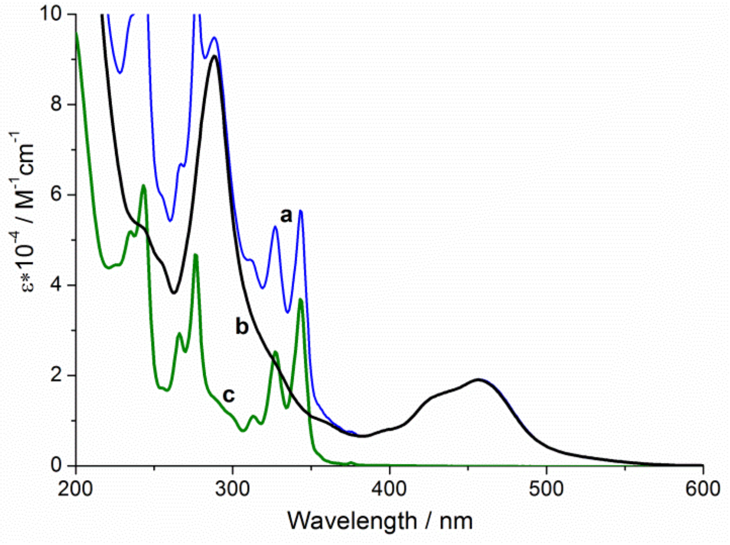

Figure S1. Electronic absorption spectra of **1** (a), **2** (b) and pyrene macrocycle **3** (c) in acetonitrile.

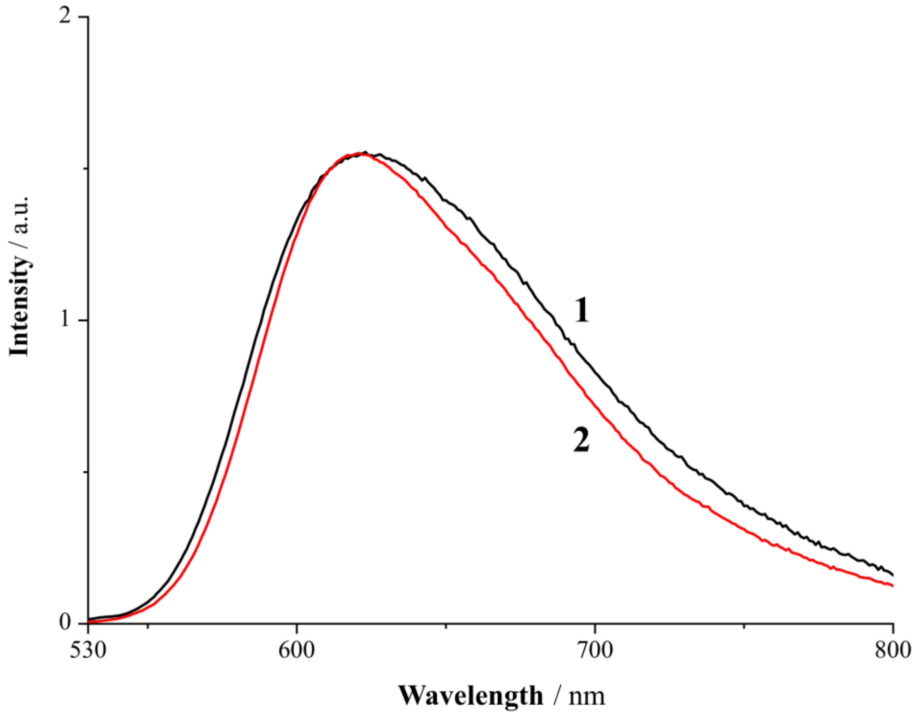

Figure S2. Photoluminescence spectra of **1** and **2** in acetonitrile (λ_{ex} = 450 nm).

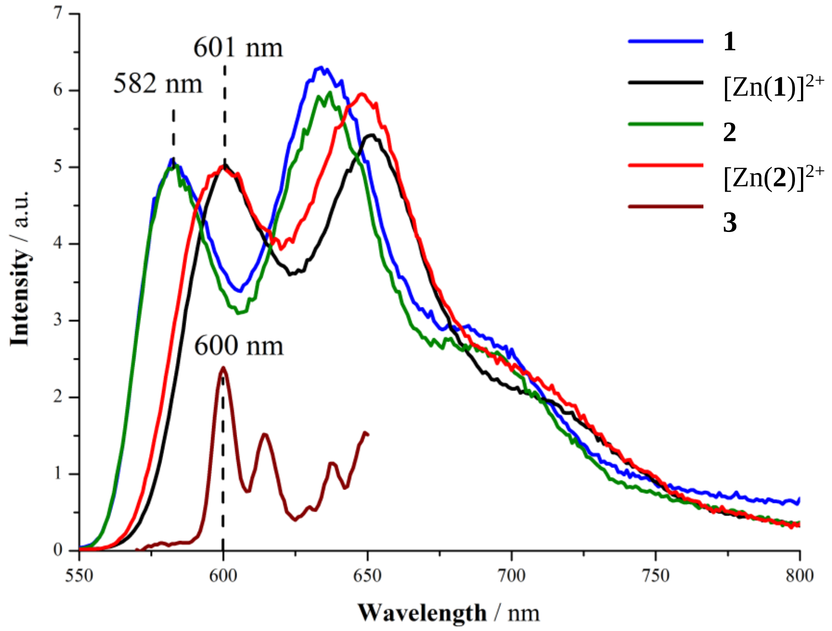

Figure S3. Phosphorescence (77 K) spectra of rotaxanes **1**, [Zn(**1**)]$^{2+}$, **2** and [Zn(**2**)]$^{2+}$ (λ_{ex} = 450 nm) and pyrene macrocycle **3** (λ_{ex} = 343 nm) in butyronitrile.

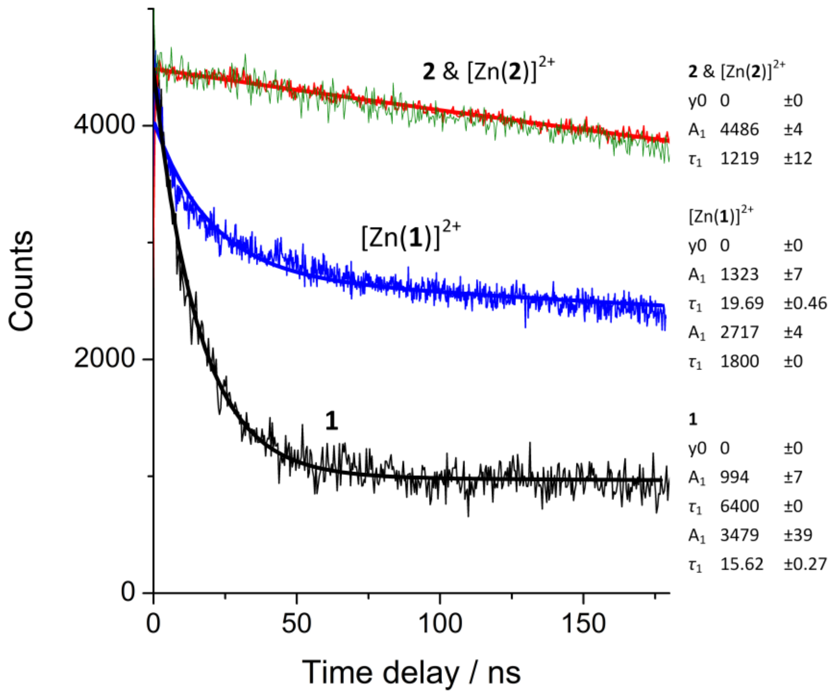

Figure S4. Fluorescence decays of rotaxanes **1**, [Zn(**1**)]$^{2+}$, **2** and [Zn(**2**)]$^{2+}$ (λ_{ex}=450 nm, λ_{em}=640 nm) in degassed acetonitrile on nanosecond time range.

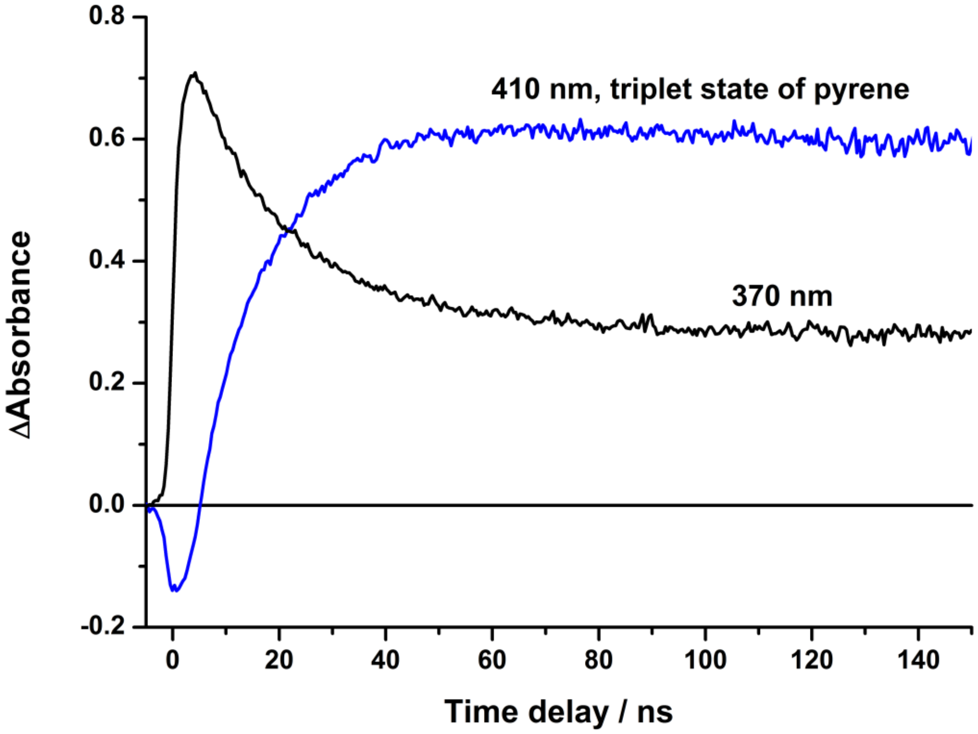

Figure S5. Transient absorption kinetics at 370 and 410 nm of rotaxane **1** in degassed acetonitrile (λ_{ex}=442 nm).

4. Detailed Photophysical Interpretation and Kinetic Analysis

The detailed photophysical behavior in rotaxane **1**, and its ion-perturbation, can be described by the interpretation outlined below, taking into account fast forward EET from excited Ru(bipy)$_3^{2+}$ towards pyrene and slow back EET, from the triplet state of slightly lower energy, as well as some conformational possibilities. Indeed, with triplet-triplet energy transfer being short range (sub-nanometric), slow interchanging chromophore conformations are considered with resulting kinetic effects.

The structure of rotaxane **1** is such that the possibility exists for pyrene to be preferentially localized at two sites by non-covalent interactions, namely pyrene close to Ru(bipy)$_3^{2+}$ complex and pyrene close to the bis(*tert*-butyl)benzene motif. Some indication that this may be the case was obtained by *ab initio* geometry optimization (MOPAC) of compound **1,** with interaction energies estimated to be similar for both sites on the order of 4 - 5 kcal/mol. In its broadest terms, we can consider the observed photophysical processes according to a 2 conformation model, namely one in which REET is effective and one in which it is not. Conformational interchange may be anticipated either in ground state or during relatively long-lived excited states. The simplified conformations **A** and **B** and corresponding schematic energy diagram of **1** is presented below:

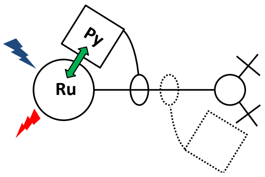

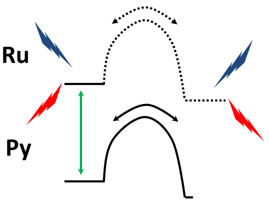

The ruthenium-complex absorbs excitation energy and emits luminescence in both **A** and **B**. REET is efficient only at the pyrene position **A**. The ratio of pyrenes at the positions **A** and **B** is defined by the energy difference of each conformation according to a Boltzmann distribution.

The dynamics of population changes corresponding to the energetic scheme and assumptions given above can be described by a system of 4 differential equations (below) corresponding to:-

1) Evolution of Ru(bipy)$_3^{2+}$ when pyrene is in position **A**. Here, the absorption, the emission of light and REET are present. The escape from position **A** towards position **B** follows the movements of the pyrene chromophore.

2) Evolution of Ru(bipy)$_3^{2+}$ when pyrene is in position **B**. Only absorption and emission of light are present. This population can return to the position **A**.

3) Pyrene when at position **A**. REET is present. Pyrene can move to **B**, overcoming the interaction energy barrier with non-photoactive stopper.

4) Pyrene when at position **B**. Only the return to position **A** is possible:

$$\begin{cases} \frac{dRu_1}{dt} = Ru_{10}G(t) - k_0 Ru_1 - k_{R\to P}Ru_1 + k_{P\to R}Py_1 - k_{1\to 2}Ru_1 + k_{2\to 1}Ru_2 \\ \frac{dRu_2}{dt} = Ru_{20}G(t) - k_0 Ru_2 + k_{1\to 2}Ru_1 - k_{2\to 1}Ru_2 \\ \frac{dPy_1}{dt} = k_{R\to P}Ru_1 - k_{P\to R}Py_1 - k_{1\to 2}Py_1 + k_{2\to 1}Py_2 \\ \frac{dPy_2}{dt} = k_{1\to 2}Py_1 - k_{2\to 1}Py_2 \end{cases}$$

The initial conditions are $R_1=R_2=P_1=P_2=0$. G(t) is the temporal profile of the excitation pulse. The excitation efficiencies of the Ru-complex at the positions 1 and 2 are $\frac{Ru_{10}}{Ru_{20}} = exp\left(\frac{-\Delta E_{12}}{kT}\right)$ and $Ru_{10} + Ru_{20} = Ru_{excited}$. The emissive relaxation rate k_0 was obtained from the luminescence measurements of the compound **2** (as well as $[Zn(\mathbf{2})]^{2+}$) and is equal to 8.3 × 10^{-7} s^{-1} (τ = 1.2 μs) independently of the temperature. All other rates are in the form $k_{i\to j} = k0_{i\to j} exp\left(\frac{-\Delta E_{i\to j}}{kT}\right)$, where $\Delta E_{i\to j}$ is the energetic barrier on the pathway from state *i* towards state *j*.

The fit of experimental kinetics for different temperatures is presented below:

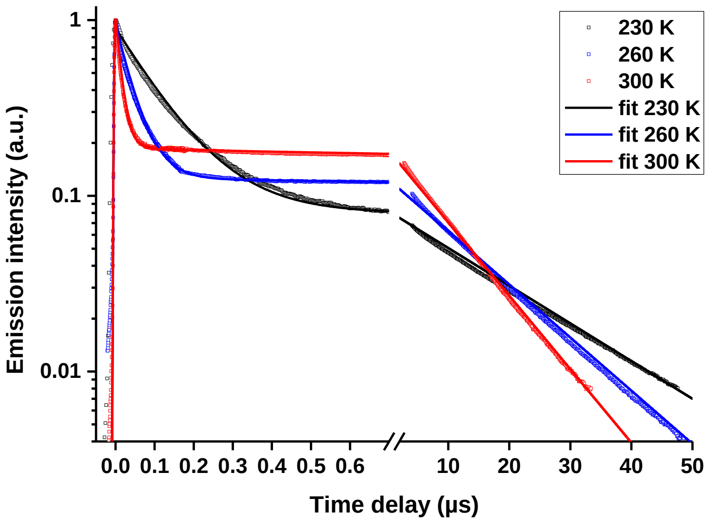

Figure S6. ^{3}MLCT emission decay over time at different temperatures and the fit of experimental kinetics.

The fit was obtained with the following parameters for 300 K: $k_{Ru\to Py}$=5.36 × 10^8 s^{-1} (τ = 1.86 ns), $k_{Py\to Ru}$=7.07 × 10^7 s^{-1} (τ = 14 ns), $k_{Py\leftrightarrow Py}$=6.68 × 10^7 s^{-1} (τ = 15 ns) and energetic barriers $\Delta E_{Py\to Ru}$=475 cm^{-1}, $\Delta E_{Py\leftrightarrow Py}$=1500 cm^{-1}. The whole system relaxes to the ground state with the rate 1.03 × 10^5 s^{-1} (τ = 9.75 μs).

The spectral shift of the emission upon complexation with Zn^{2+} is 705 cm^{-1}. This suggests that the energetic scheme for the [Zn(**1**)]$^{2+}$ complex should be similar to that of **1**, except the $Ru(bpy)_3^{2+}$ ^{3}MLCT level is shifted down by 705 cm^{-1}. Having in mind that the energy difference between Py and Ru obtained from previous fit is equal to 475 cm^{-1}, the energy difference between Ru and Py in the complex should be 230 cm^{-1}:

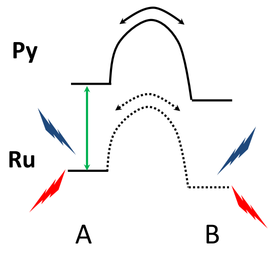

The fit using adapted for the new energetic configuration set of differential equations produces the values at 300 K $k_{Ru\rightarrow Py}=1.33\times10^8$ s^{-1} (τ = 7.5 ns) and $k_{Py\rightarrow Ru}=2.7\times10^8$ s^{-1} (τ = 3.7 ns). The whole system relaxes to the ground state with the rate 5.7×10^5 s^{-1} (τ = 1.75 μs).

5. Zinc-induced Spectral & Dynamics Changes

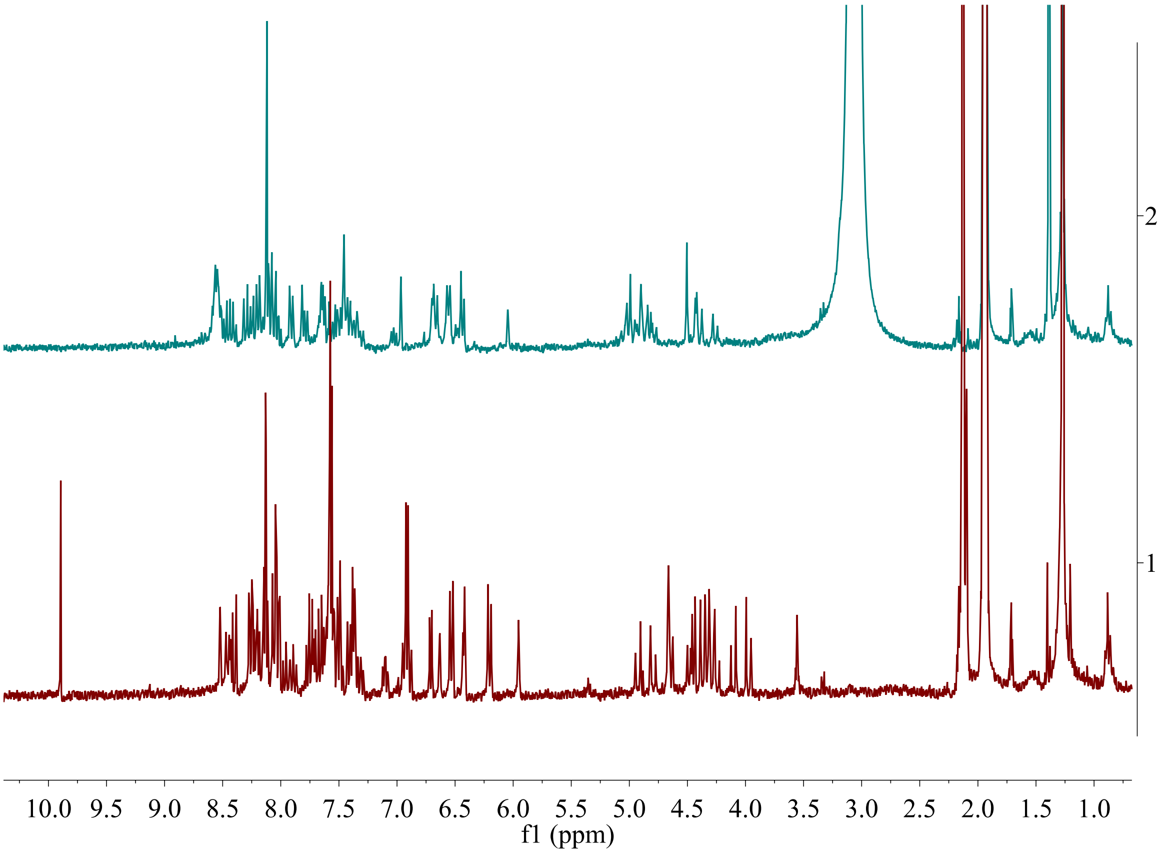

Figure S7. ^{1}H NMR spectra of rotaxane **1** (bottom) and [Zn(**1**)]$^{2+}$ (4 equiv. of $Zn(ClO_4)_2{\cdot}6H_2O$**,** top) in CD_3CN.

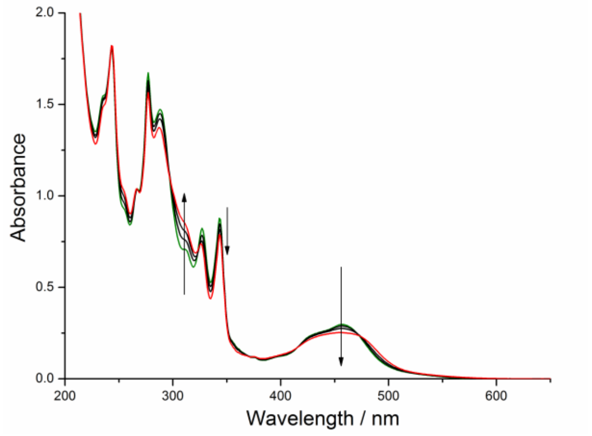

Figure S8. Electronic absorption spectra of **1** on adding $Zn(ClO_4)_2$ in air equilibrated acetonitrile. (No. of equivalents of Zn^{2+} in order of increasing absorbance at 310 nm : 0, 0.4, 0.8, 4.5). (Corresponding luminescence spectra in Figure 4).

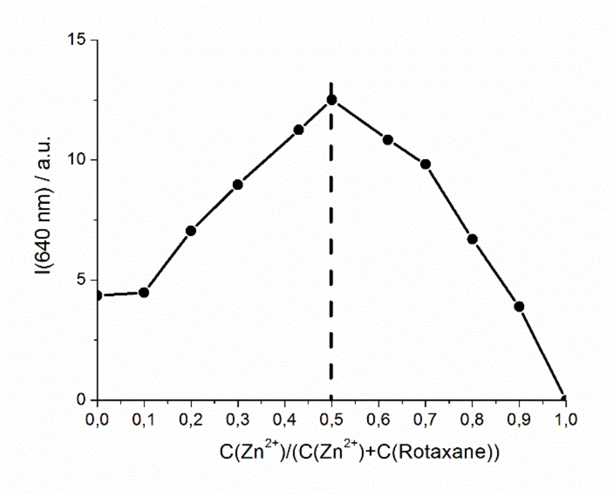

Figure S9. Luminescence Job plot of rotaxane **1** in acetonitrile (λ_{em}=640 nm). Initial C(**1**)=1.8×10^{-5} M.

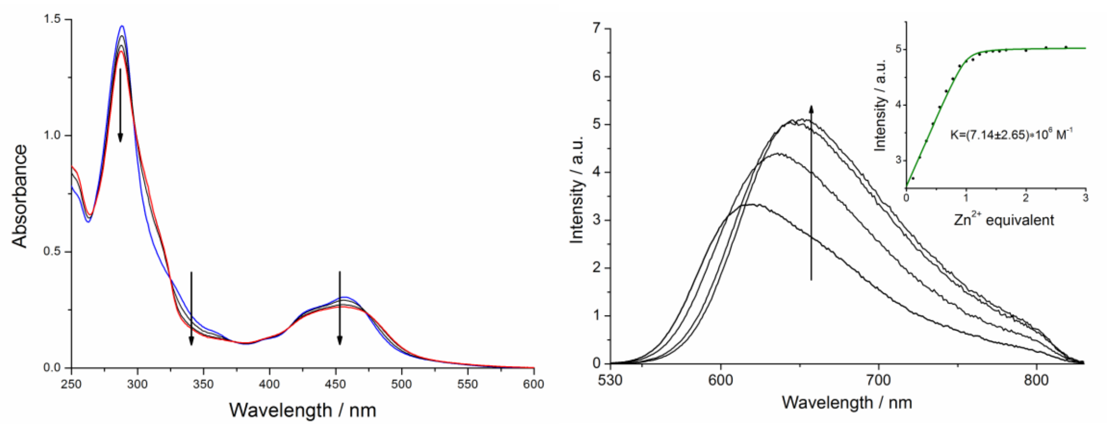

Figure S10. Electronic absorption and luminescence spectra (λ_{ex} = 475 nm) of **2** on titrating with $Zn(ClO_4)_2$ in acetonitrile. C(**2**)=1.52×10^{-5} M; Equivalents of added Zn^{2+} following arrows in electronic absorption and luminescence spectra = 0, 0.5, 1, 1.5. Inset: Change in luminescence intensity at 660 nm on adding of $Zn(ClO_4)_2{\cdot}6H_2O$.

Contributions in absorption and luminescence for **1** and **2** at a given wavelength is a sum of contributions from rotaxane and rotaxane bound with Zn^{2+}. Taking that into account, the binding constant can be determined from experimental findings according to approximation :-[S5]

$$I_{total} = I_L + \frac{I_{LM} - I_L}{2C_L}\left(C_M + C_L + \frac{1}{K} - \sqrt{\left(C_M + C_L + \frac{1}{K}\right)^2 - 4C_LC_M}\right)$$

I_{total} is total intensity (λ = 640 nm), I_L is initial luminescence intensity of rotaxane, I_{LM} is luminescence intensity at the end of the titration, C_L is initial concentration of rotaxane, C_M - concentration of $Zn(ClO_4)_2$ added, K is binding constant.[S6]

Table S1. Quantum yields (Φ) of **1**, [Zn(**1**)]$^{2+}$, **2**, [Zn(**2**)]$^{2+}$ and luminescence lifetimes in acetonitrile at room temperature.

	Φ^{degas}	Φ^{air}	τ_1 / ns	τ_2 / μs
**1**	0.11	0.006	16	9.75
[Zn(**1**)]$^{2+}$	0.13	0.030	20 (13)	1.8
**2**	0.11	0.022	-	1.1
[Zn(**2**)]$^{2+}$	0.13	0.032	-	1.2

Short lifetimes (τ_1) for **1** and [Zn(**1**)]$^{2+}$ are obtained from treatment in frame of two exponential decay model of the kinetics in nanosecond time range (**Figure 2**). Second times τ_2 were obtained from the fitting of kinetics in microsecond time range (**Figure S3**) of one exponential decay and were taken as given from fitting of kinetics in nanosecond time range. The reference compound is $[Ru(bpy)_3]Cl_2$ in H_2O (bpy=2,2'-bipyridine) (Φ_{em} = 0.028).[S7]

For rotaxane **1** and [Zn(**1**)]$^{2+}$ values in the third column (Table S1) are obtained from transient absorption kinetics at 410 nm of rotaxane (Figure S3).

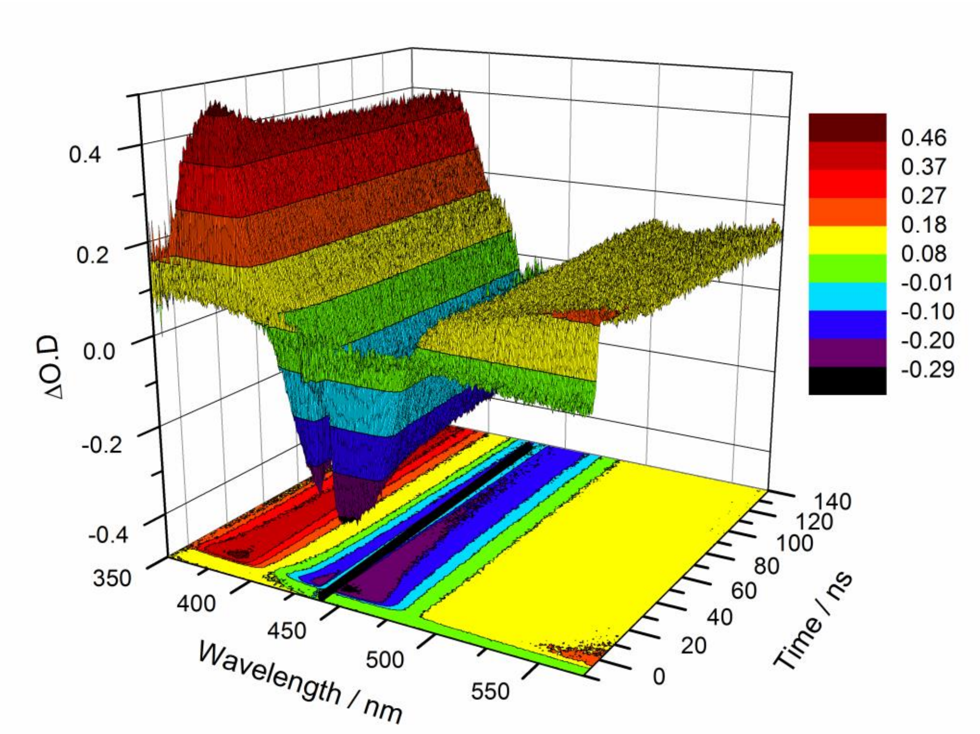

Figure S11. Transient absorption map showing energy redistributing between ring and stopper in rotaxane [Zn(**1**)]$^{2+}$. Black rectangle in 2D graph is trace from filter 442 nm.

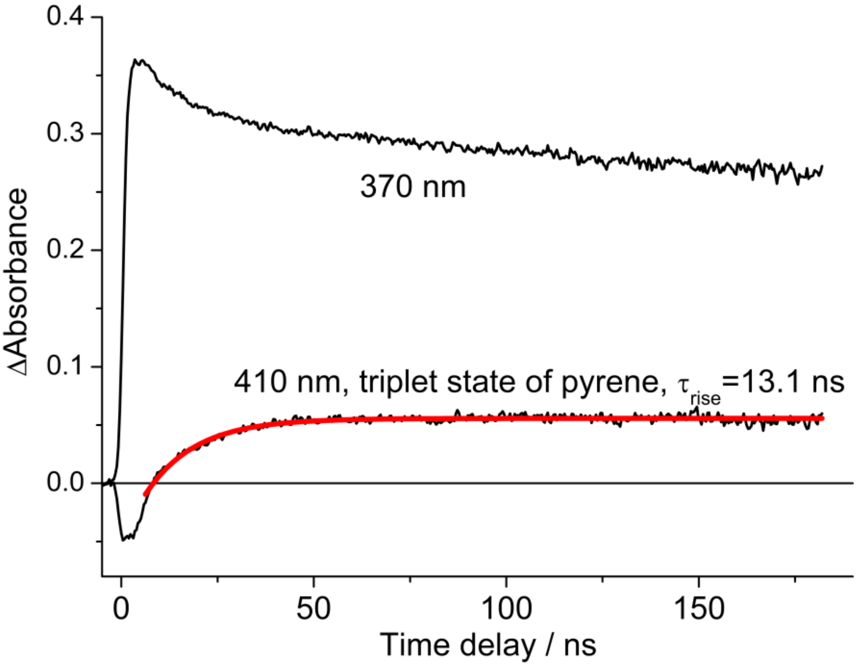

Figure S12. Transient absorption kinetics at 370 and 410 nm of $[Zn(\mathbf{1})]^{2+}$ in degassed acetonitrile (λ_{ex} = 442 nm). The kinetic at 370 nm corresponds to superposition of decrease of ruthenium complex in ^{3}MLCT state and grow-in of pyrene in triplet state. The kinetic at 410 nm corresponds to grow-in of pyrene in triplet state (red solid line = fitting). Rise time of kinetics at 410 nm is 13.1 ns.

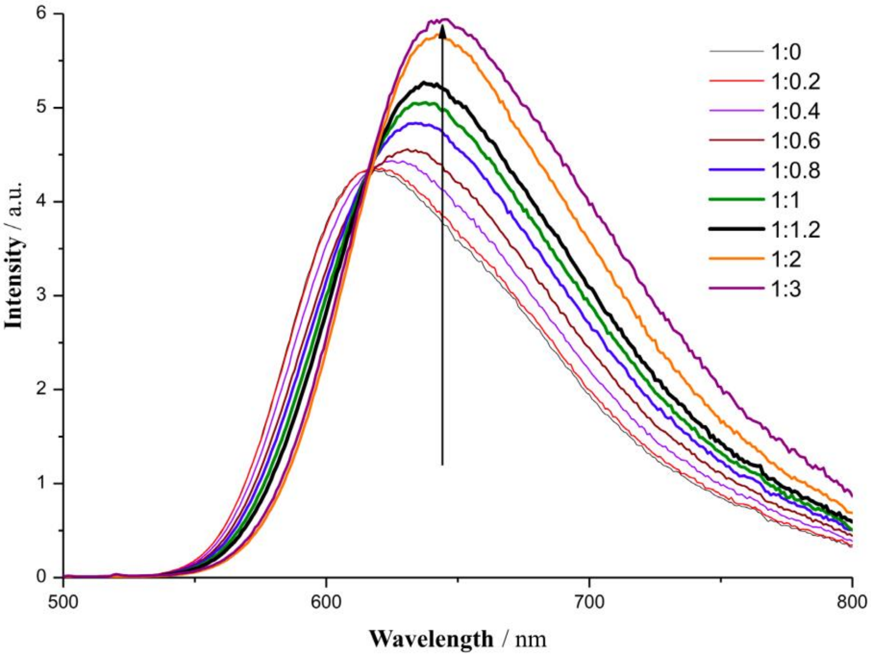

Figure S13. Luminescence emission titration of rotaxane **1** in degassed MeCN (1.18×10^{-5} M, λ_{ex} = 477 nm) with $Zn(ClO_4)_2{\cdot}6H_2O$ (0, 0.2, 0.4, 0.6, 0.8, 1.0, 1.2, 2.0, 3.0 eq).

6. NMR spectra

2-Bromo-6-((4-chloromethyl-benzyloxy)methyl)pyridine (8)

^{1}H NMR (300 MHz $CDCl_3$)

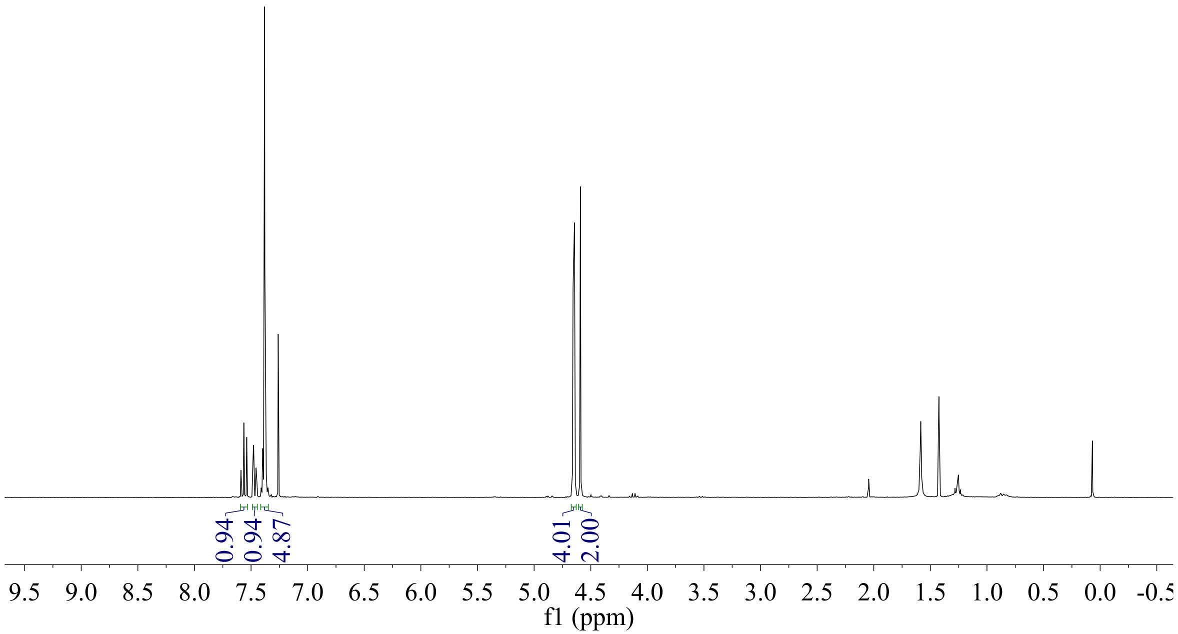

^{13}C NMR (75 MHz $CDCl_3$)

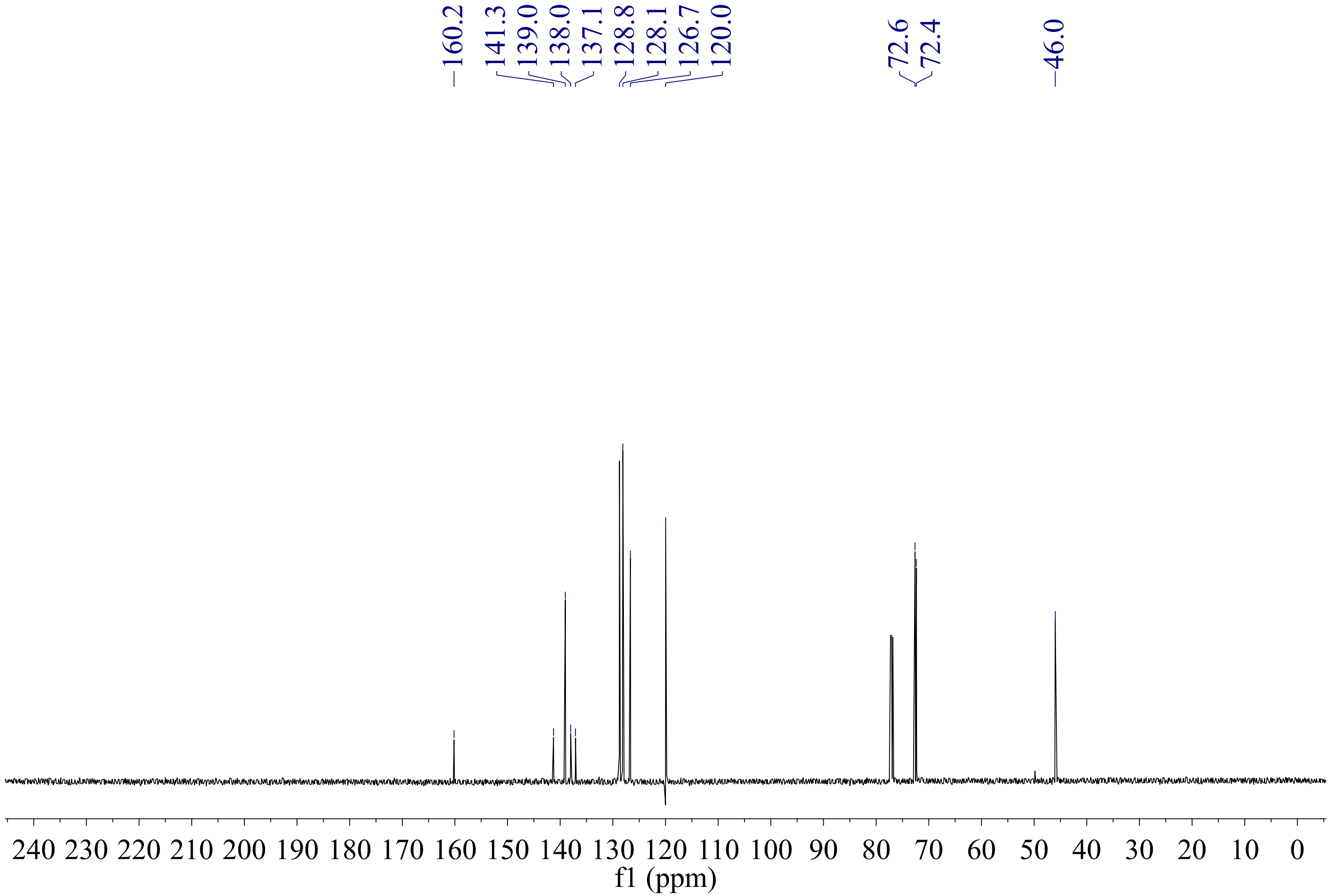

(3,5-dimethyoxylphenyl)pyren-1-ylmethanol (9)

^{1}H NMR (300 MHz $CDCl_3$)

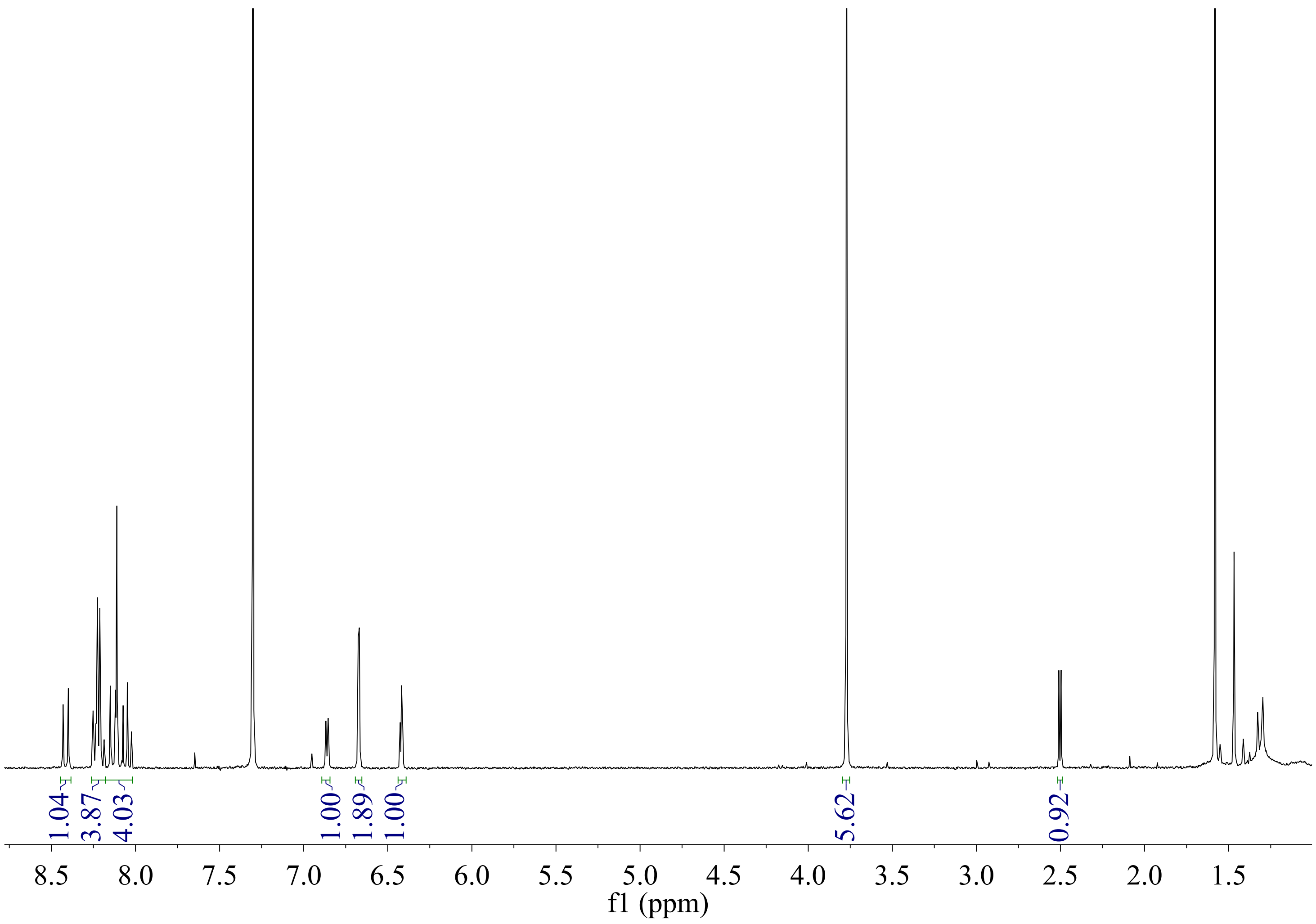

^{13}C NMR (75 MHz CDCl$_3$)

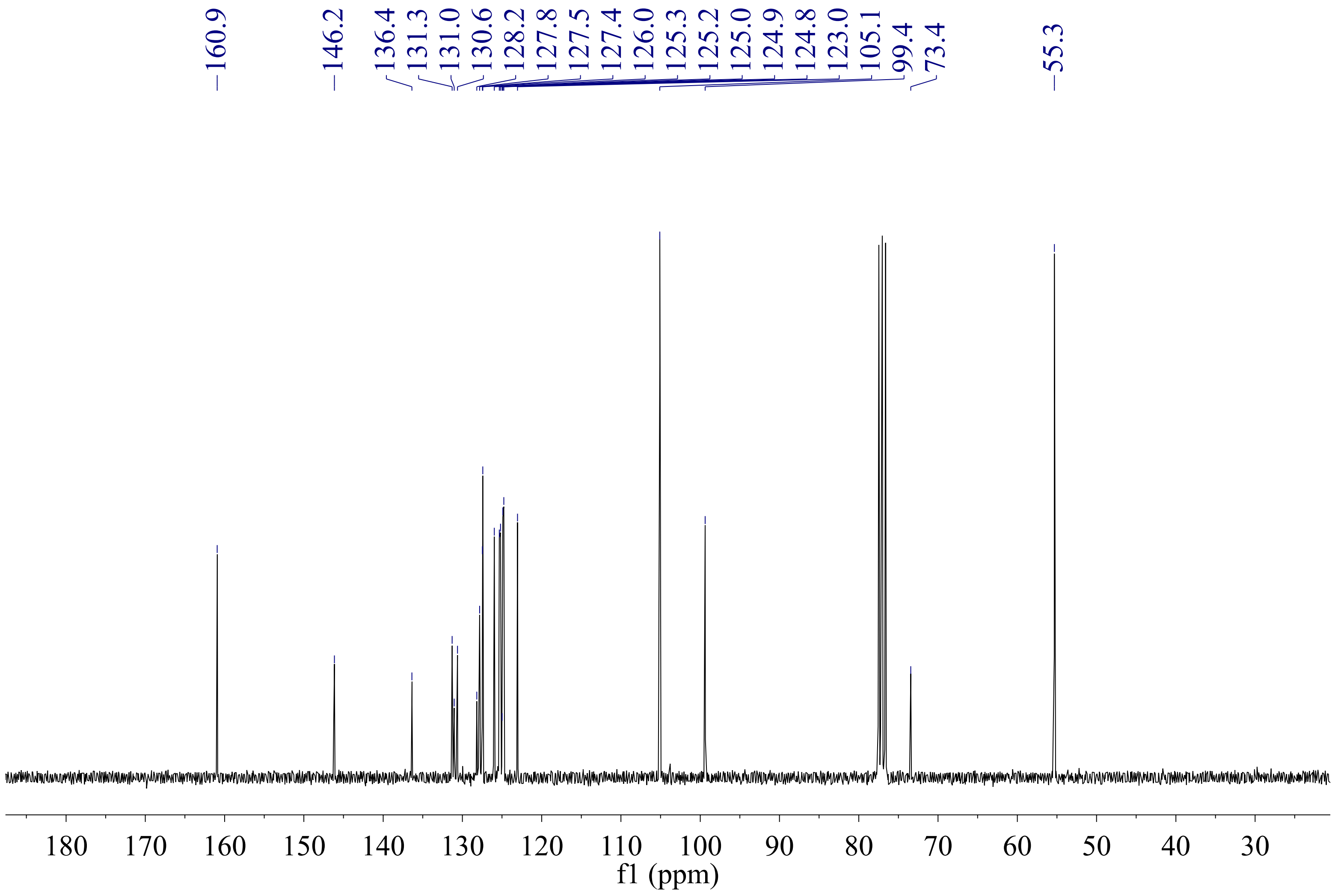

1-(3,5-dimethyoxylbenzyl)pyrene (10)

^{1}H NMR (300 MHz $CDCl_3$)

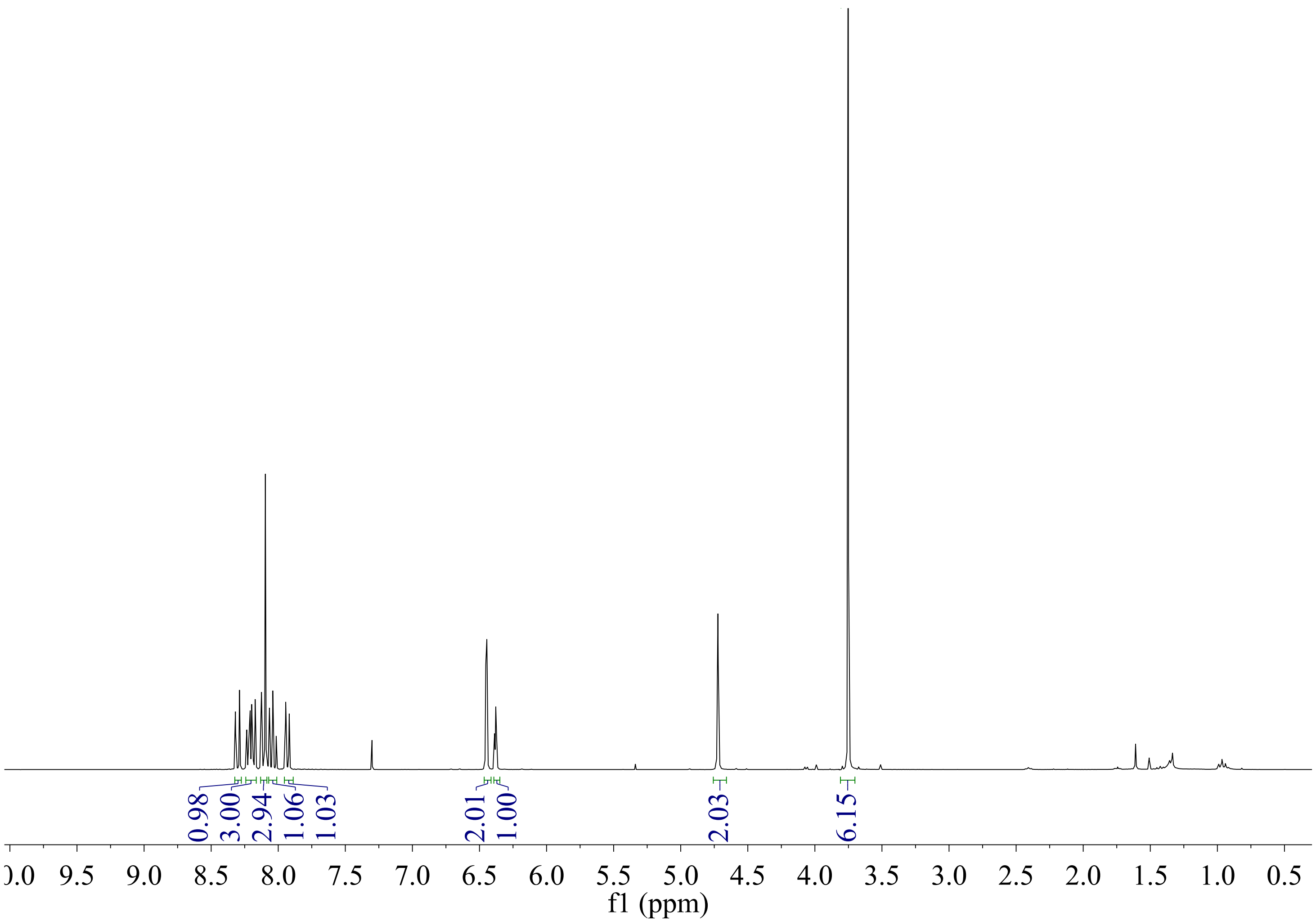

^{13}C NMR (75 MHz $CDCl_3$)

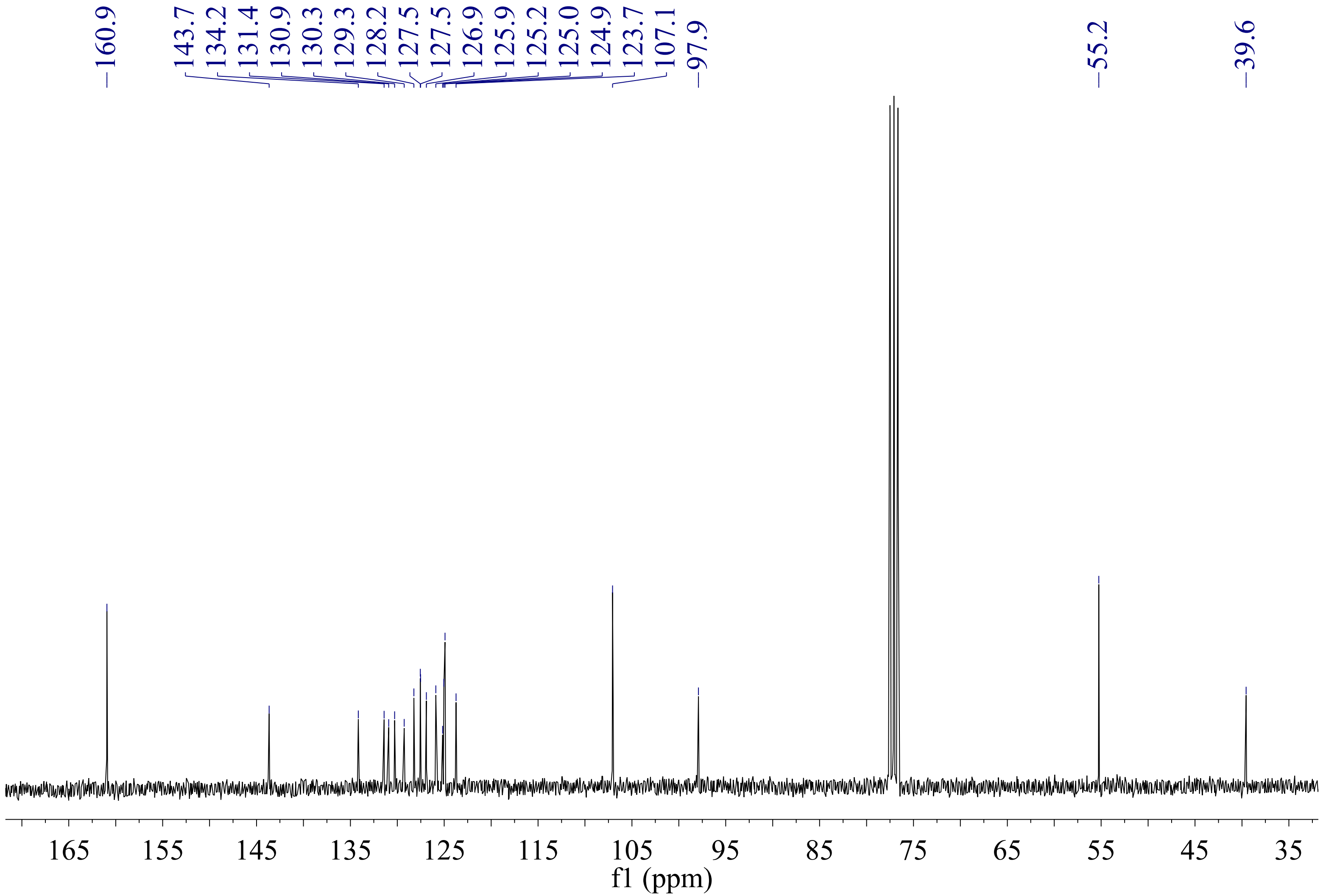

1-(3,5-dimethanolbenzyl) pyrene (11)

^{1}H NMR (300 MHz $CDCl_3$)

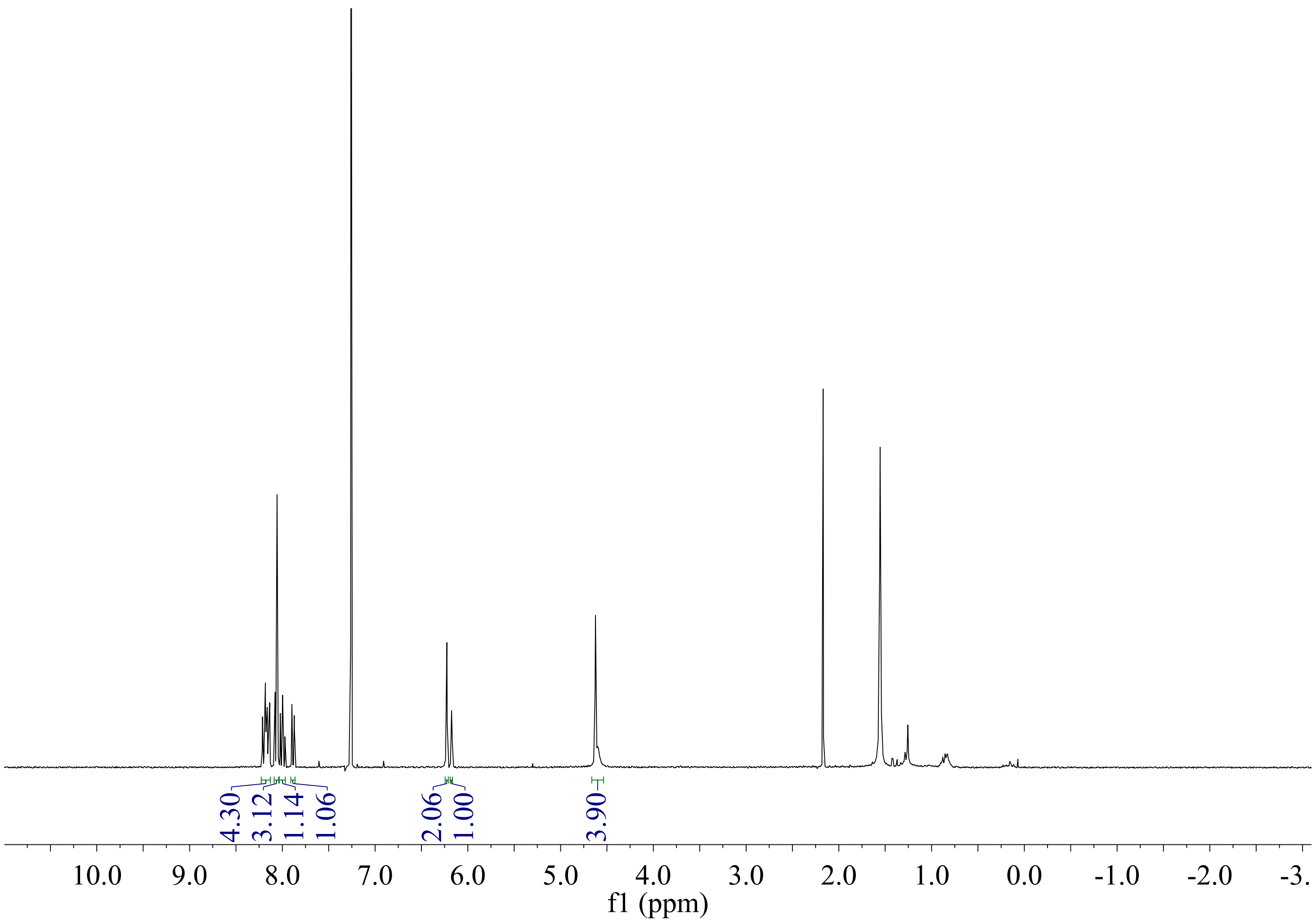

^{13}C NMR (75 MHz Tetrahydrofuran-d_8)

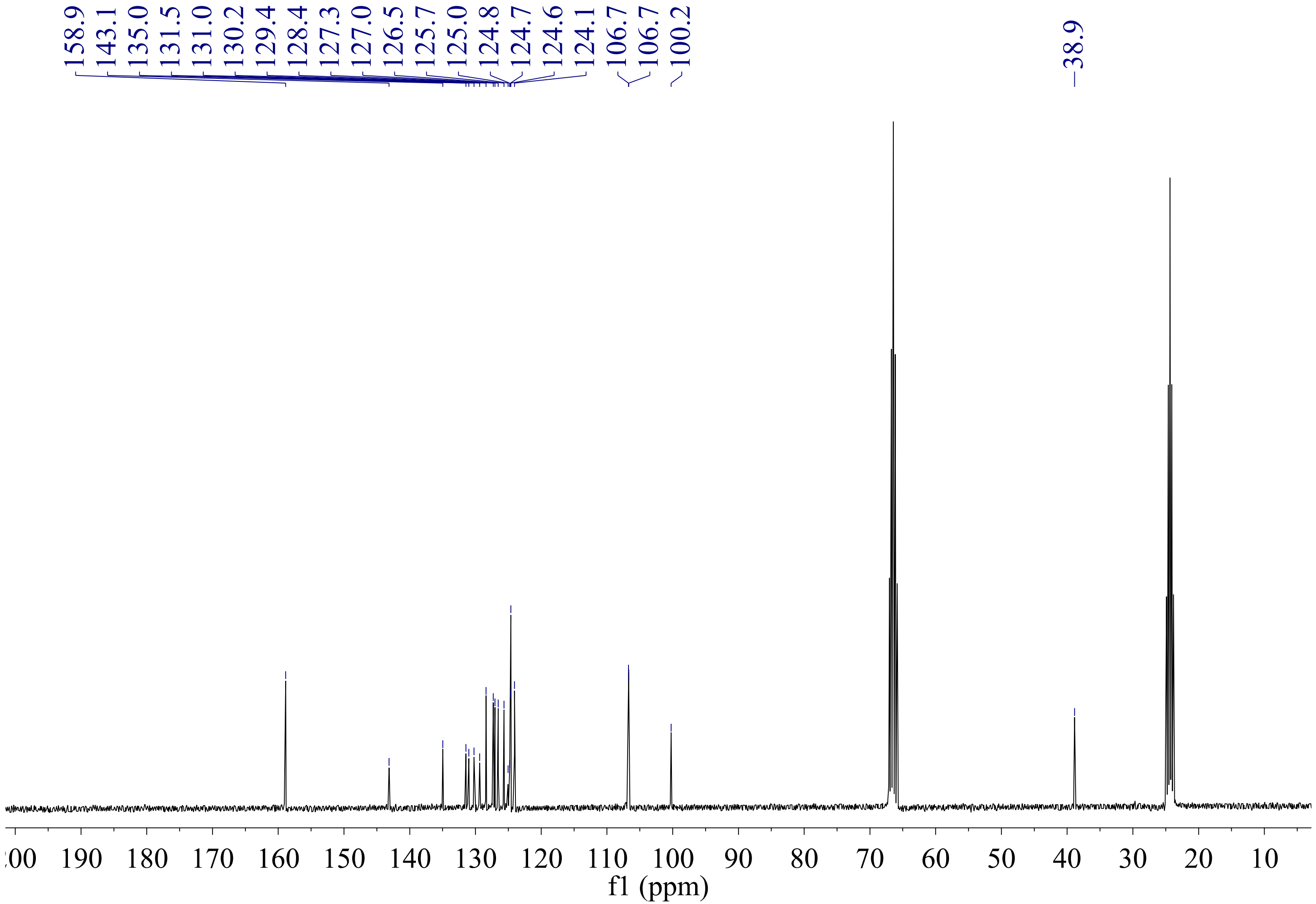

Pyrene-macrocycle precursor 12

^{1}H NMR (300 MHz $CDCl_3$)

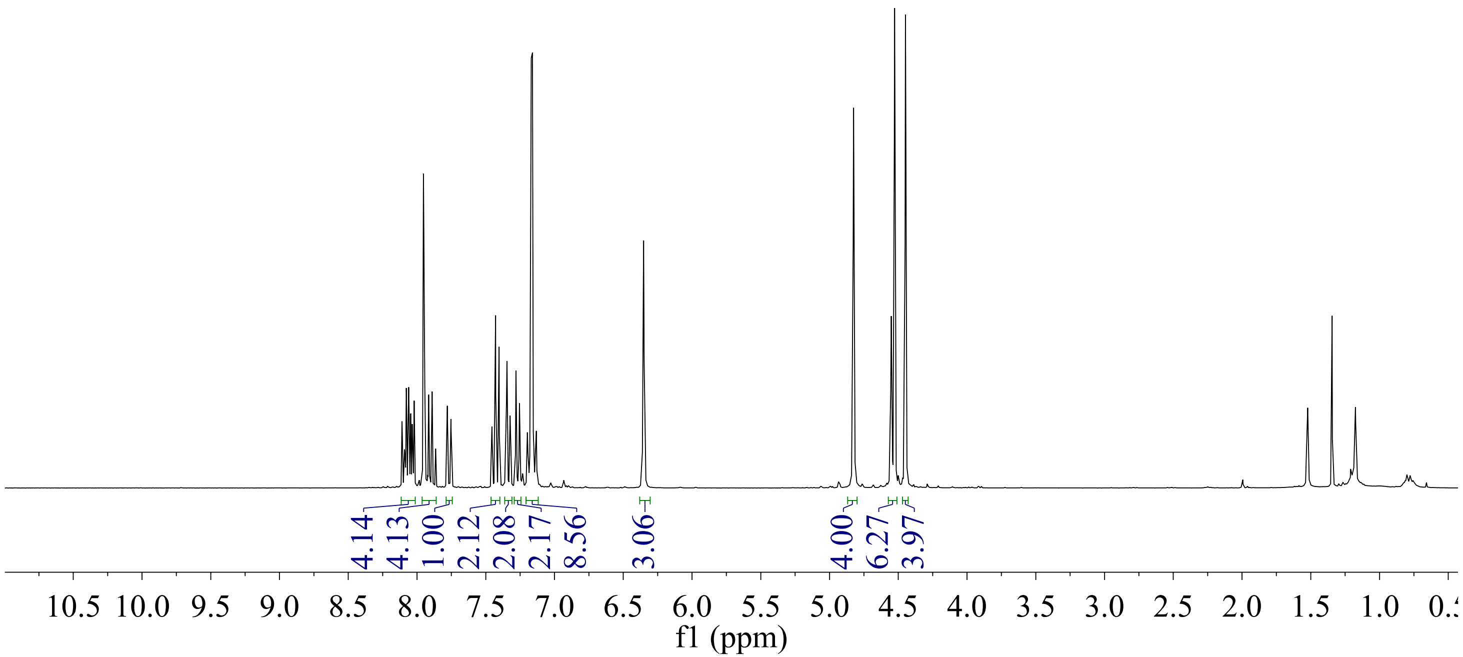

^{13}C NMR (75 MHz $CDCl_3$)

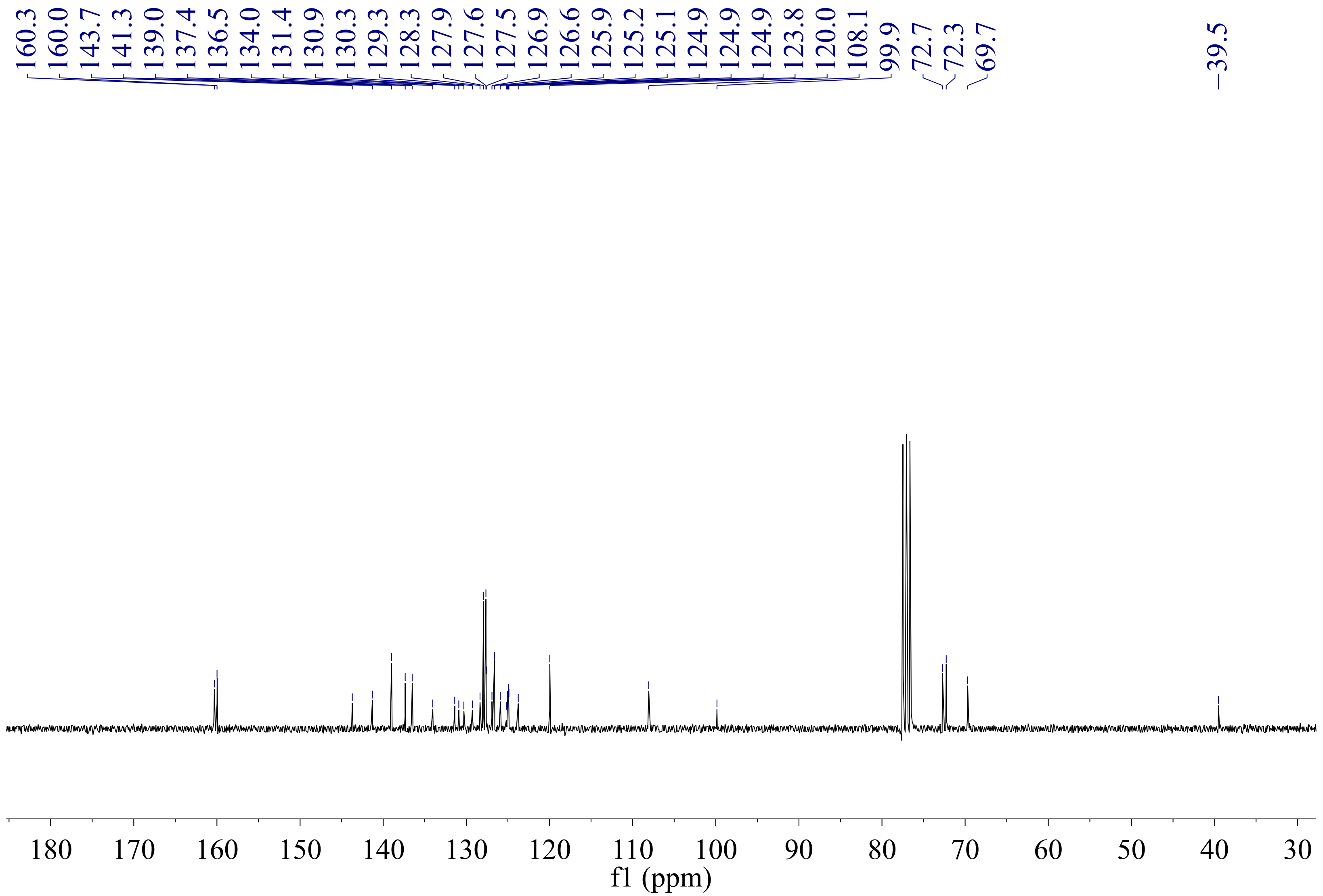

Pyrene-macrocycle 3

^1H NMR (300 MHz $CDCl_3$)

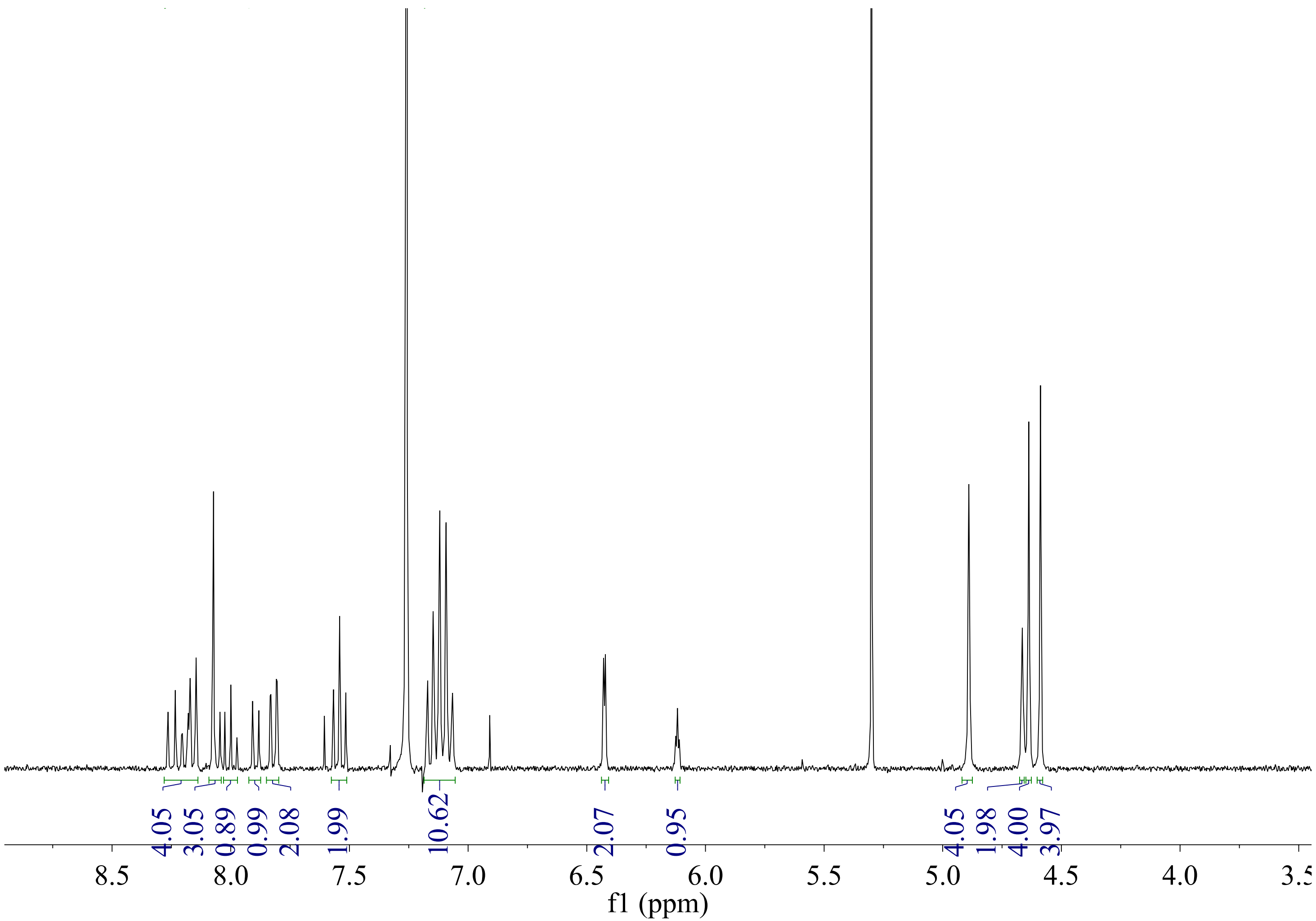

^{13}C NMR (75 MHz $CDCl_3$)

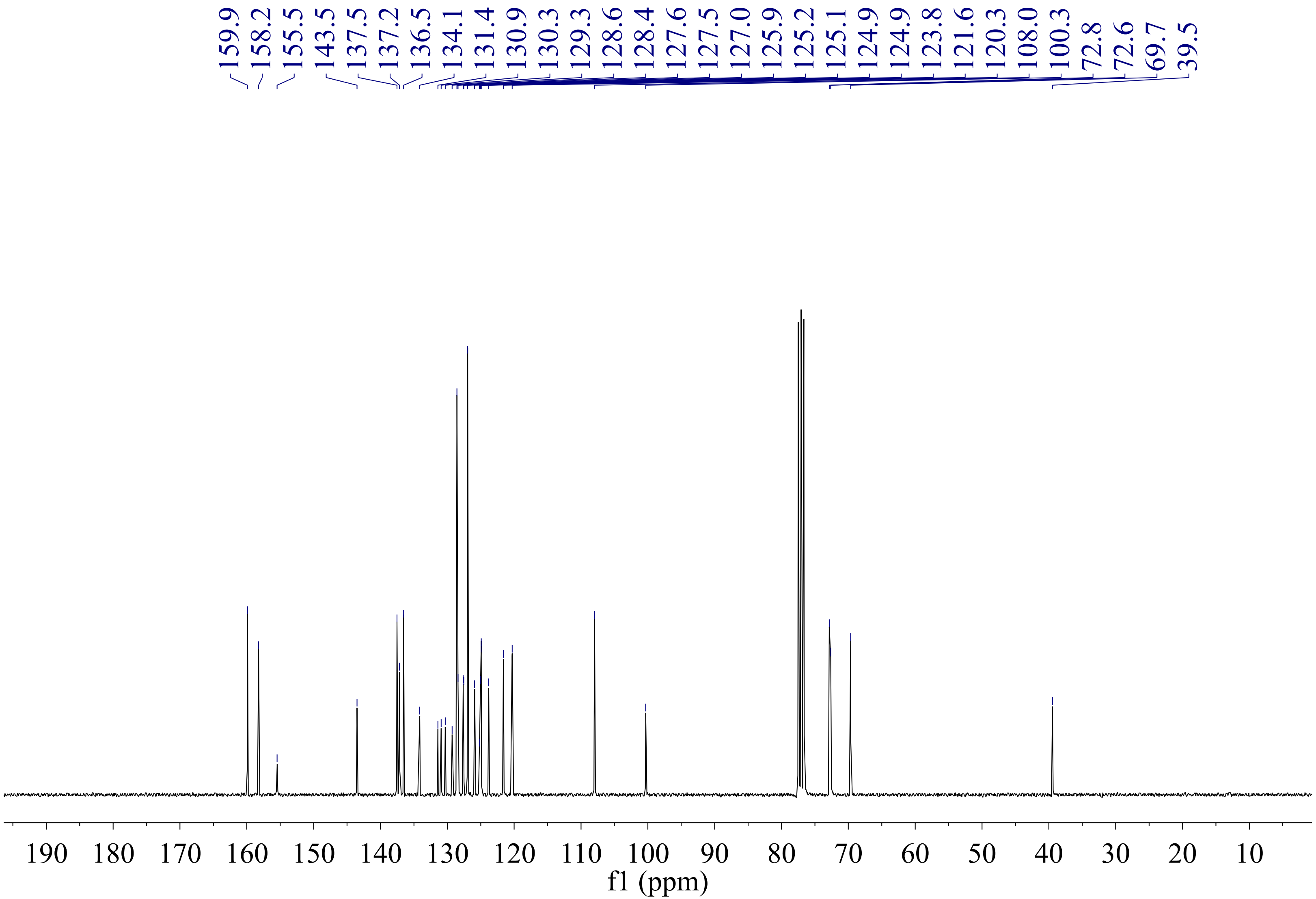

Macrocycle precursor 13

^{1}H NMR (300 MHz $CDCl_3$)

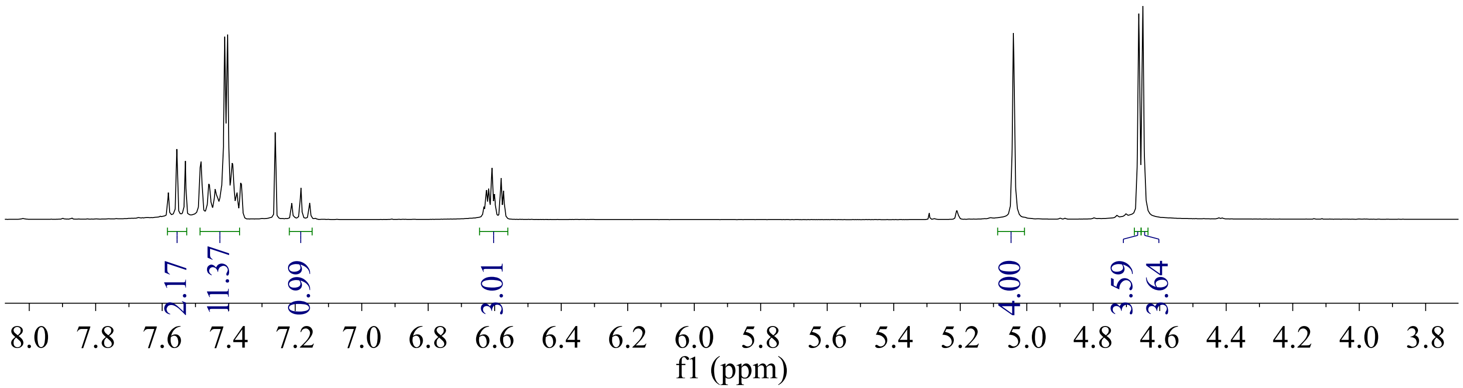

^{13}C NMR (75 MHz $CDCl_3$)

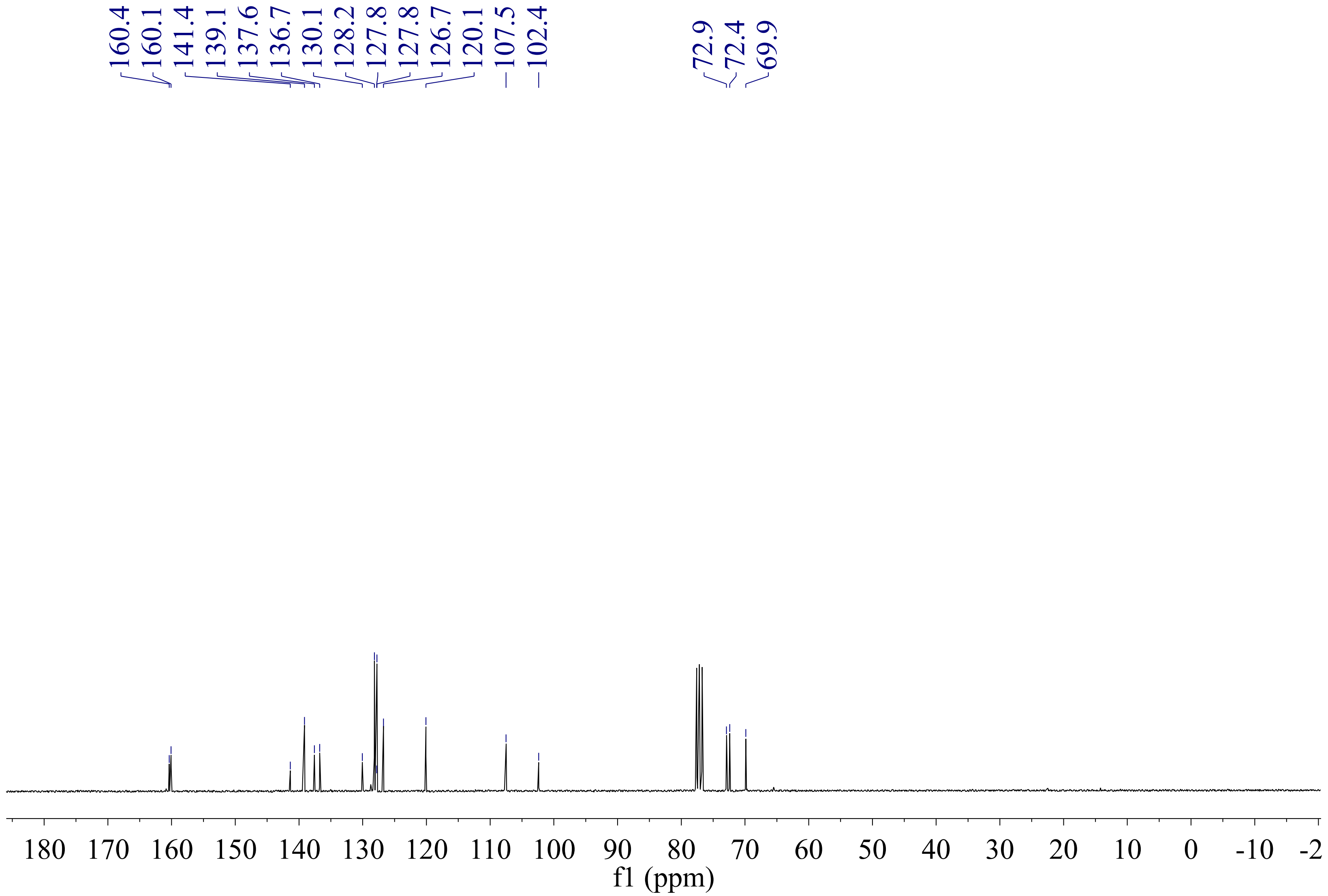

Macrocycle 4

^{1}H NMR (300 MHz CD_3CN)

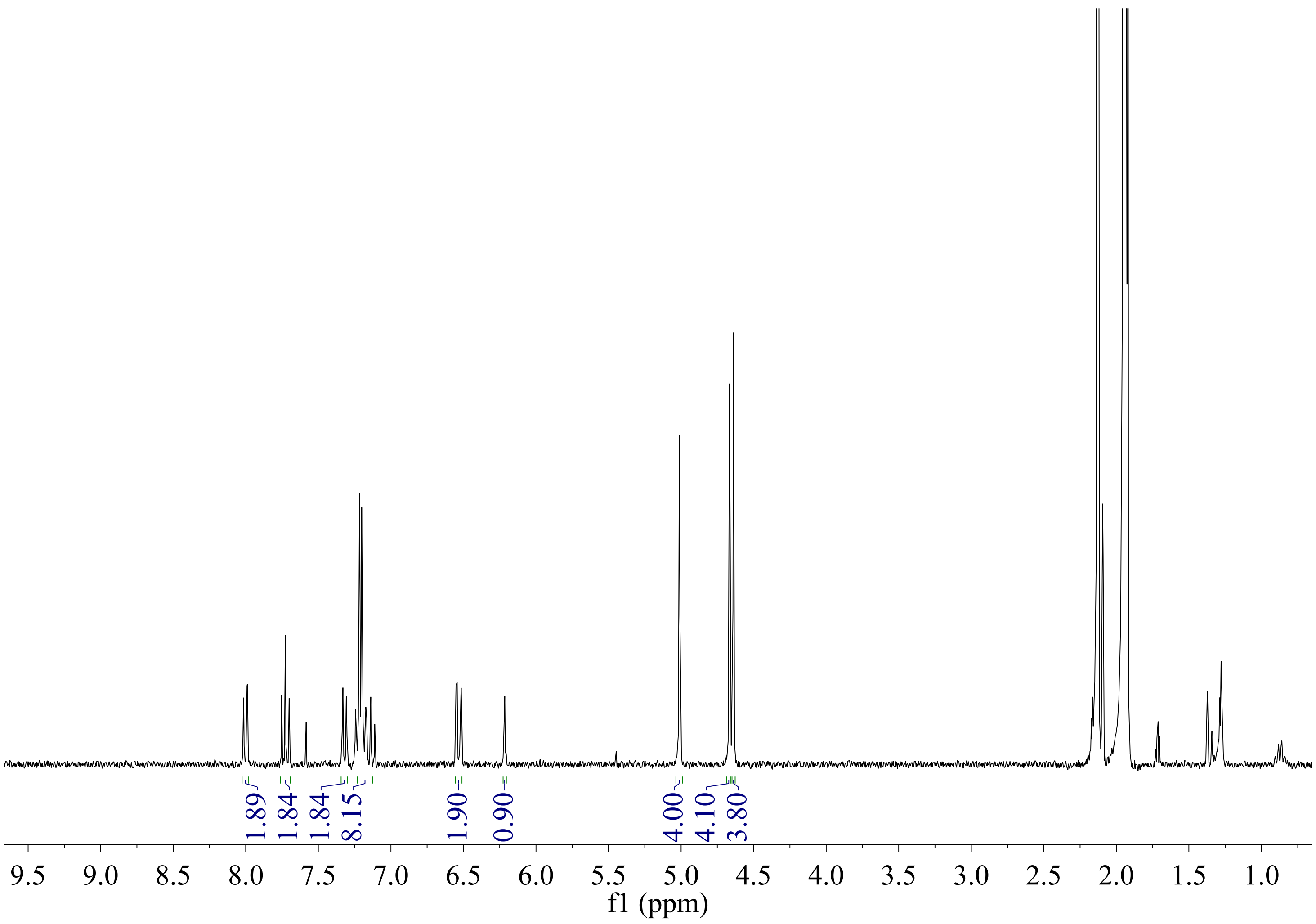

^{13}C NMR (75 MHz $CDCl_3$)

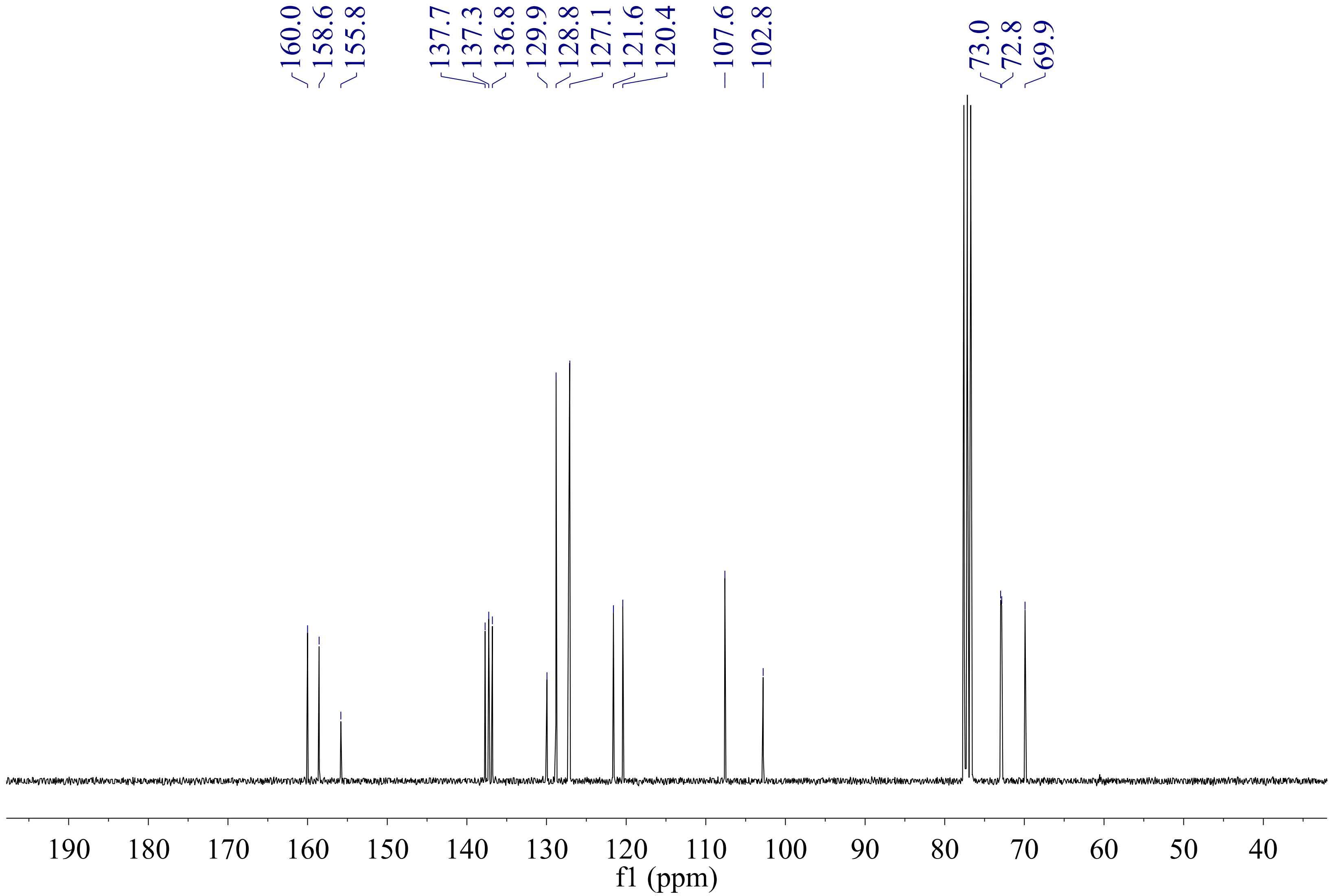

Rotaxane 1

^{1}H NMR (300 MHz CD_3CN)

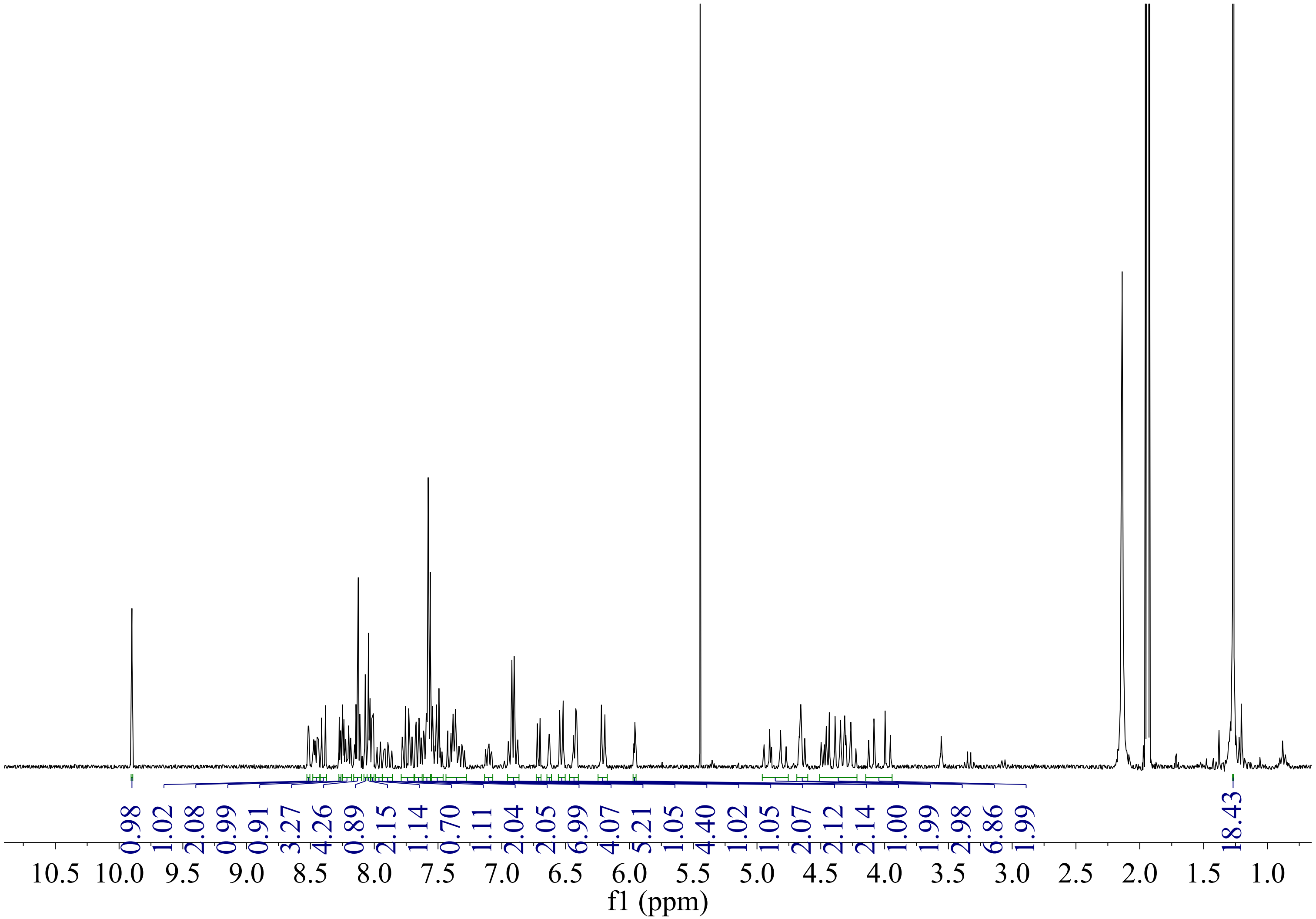

^{13}C NMR (150 MHz CD_3CN)

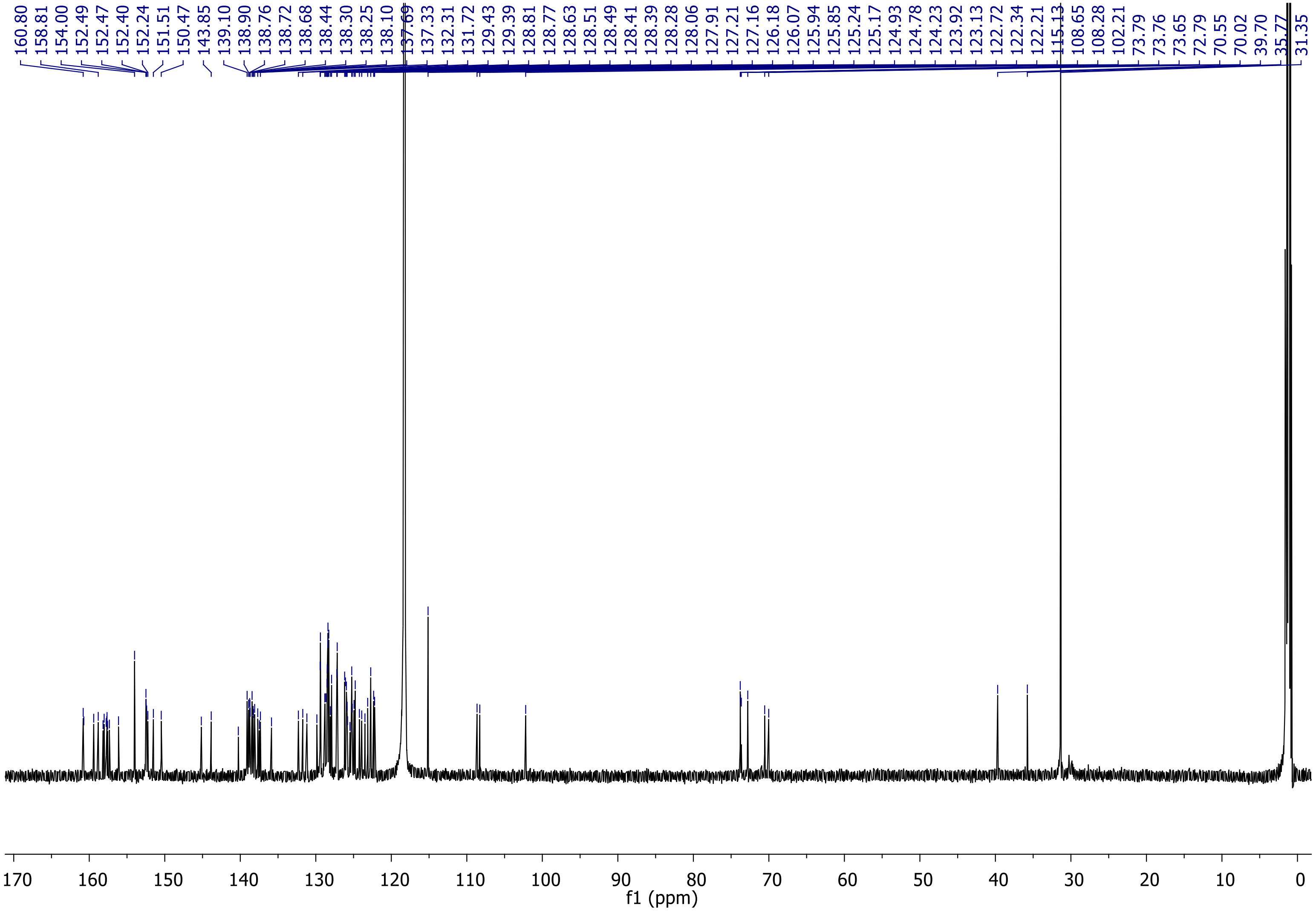

Rotaxane 2

^{1}H NMR (300 MHz CD_3CN)

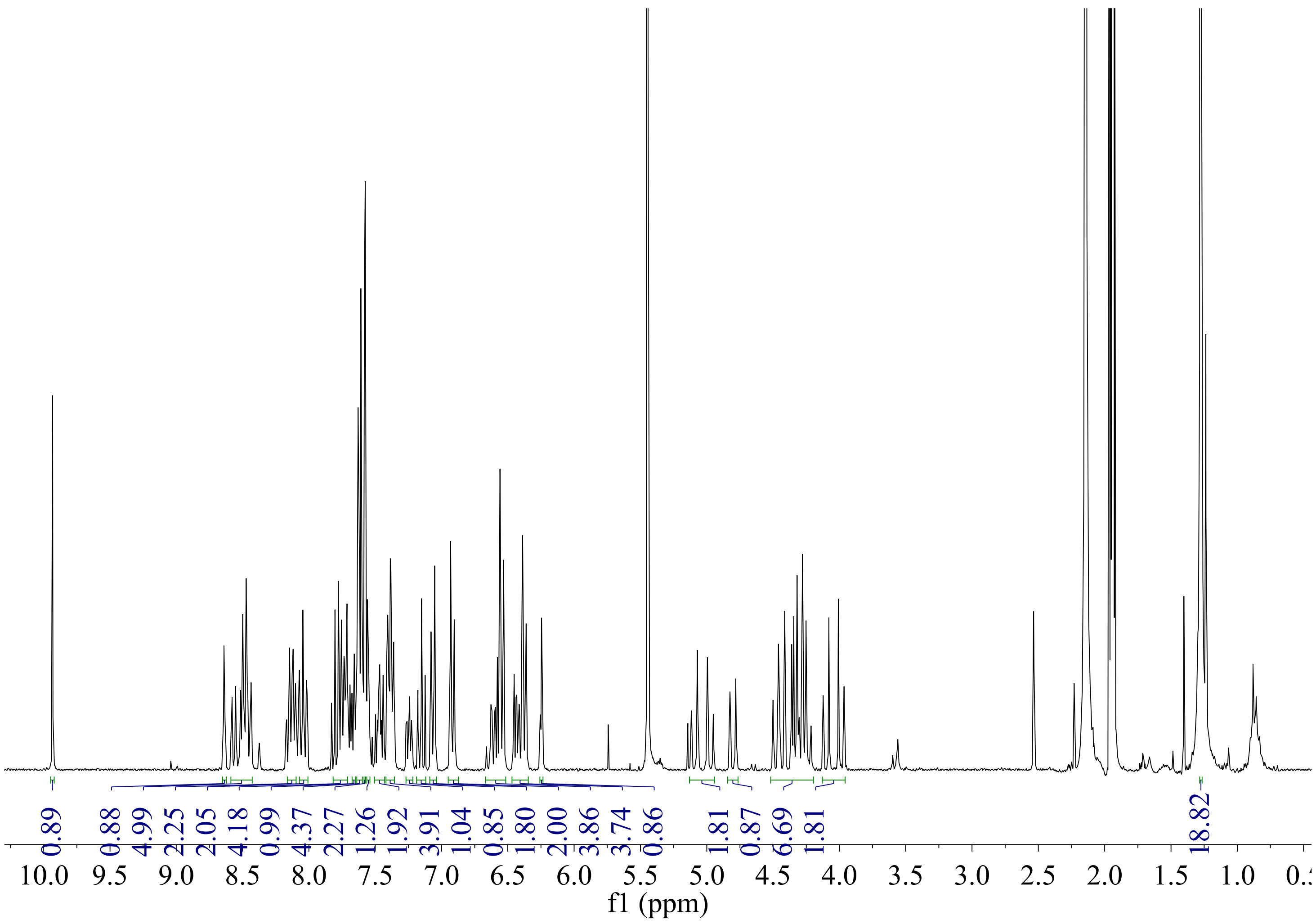

^{13}C NMR (150 MHz CD_3CN)

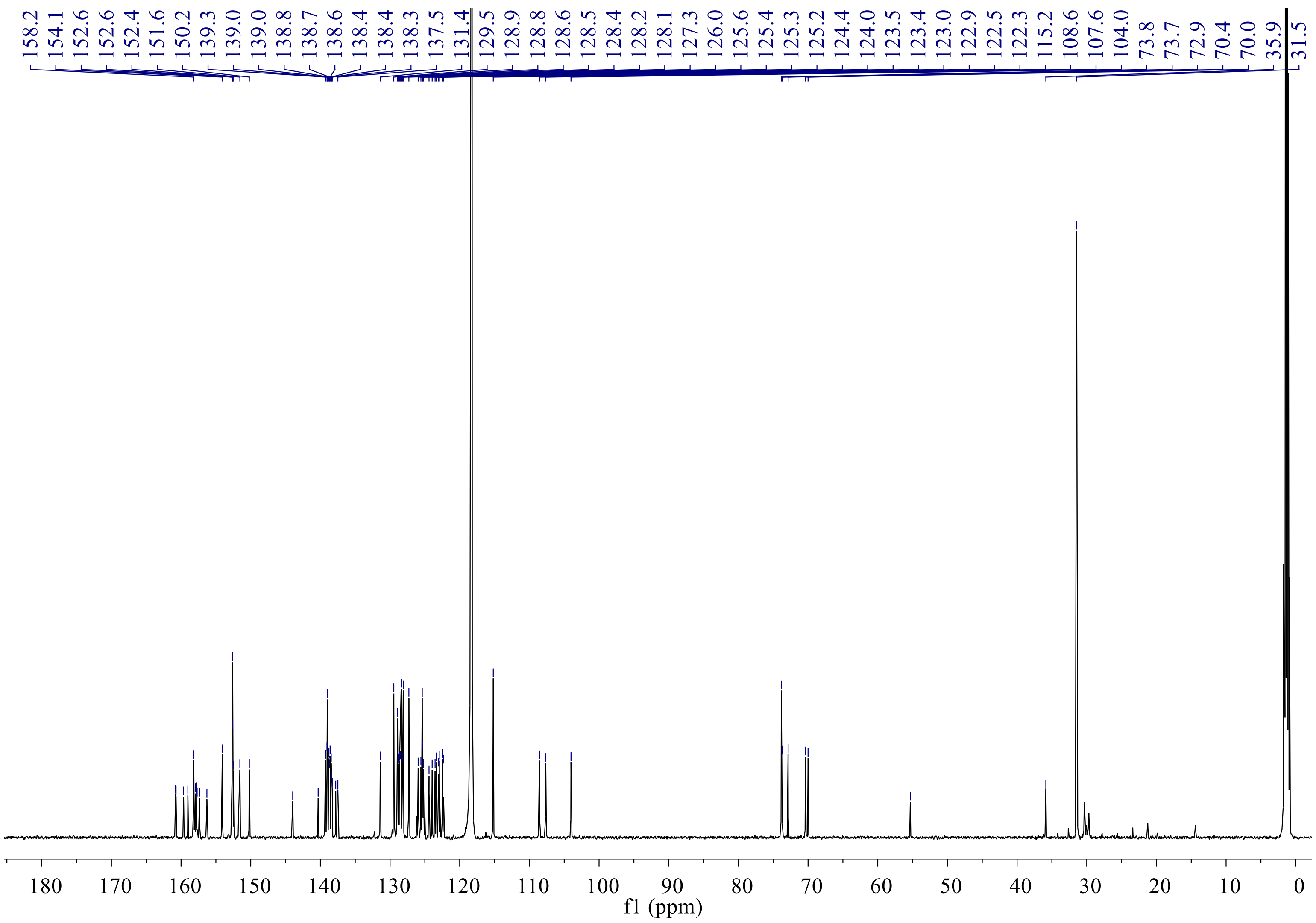

7. References

[S1] E. M. Schuster, M. Botoshansky and M. Gandelman, *Angew. Chem. Int. Ed.* 2008, **47**, 4555.

[S2] A. Baron, C. Herrero, H. Quaranta, M.-F. Charlot, W. Leibl, B. Vauzeilles and A. Aukauloo, *Inorg. Chem.* 2012, **51**, 5985.

[S3] J. E. M. Lewis, R. J. Bordoli, M. Denis, C. J. Fletcher, M. Galli, E. A. Neal, E. M. Rochette and S. M. Goldup *Chem. Sci.* 2016, **7**, 3154.

[S4] H. Ishida, S. Tobita, Y. Hasegawa, R. Katoh and K. Nozaki, *Coord. Chem. Rev.* 2010, **254**, 2449.

[S5] K. Nakamaru, *Bull. Chem. Soc. Jpn.* 1982, **55**, 2697.

[S6] K. A. Connors, Binding Constants: The Measurement of Molecular Complex Stability. *New York: John Wiley & Sons*, 1987.

[S7] J. Bourson, J. Pouget and B. Valeur, *J. Phys. Chem*. 1993, **97**, 4552.
